# Emergent tetratic order in crowded systems of rotationally asymmetric hard kite particles

**DOI:** 10.1038/s41467-020-15723-w

**Published:** 2020-04-28

**Authors:** Zhanglin Hou, Yiwu Zong, Zhaoyan Sun, Fangfu Ye, Thomas G. Mason, Kun Zhao

**Affiliations:** 1 Key Laboratory of Systems Bioengineering (Ministry of Education), School of Chemical Engineering and Technology, Tianjin University, 300072 Tianjin, P. R. China; 2 State Key Laboratory of Polymer Physics and Chemistry, Changchun Institute of Applied Chemistry, Chinese Academy of Sciences, 130022 Changchun, P. R. China; 3 University of Science and Technology of China, 230026 Hefei, P. R. China; 4 Beijing National Laboratory for Condensed Matter Physics and Laboratory of Soft Matter and Biological Physics, Institute of Physics, Chinese Academy of Sciences, 100190 Beijing, P. R. China; 5 Wenzhou Institute, University of Chinese Academy of Sciences, 325001 Wenzhou, P. R. China; 6 Department of Physics and Astronomy, University of California Los Angeles, Los Angeles, CA 90095 USA; 7 Department of Chemistry and Biochemistry, University of California Los Angeles, Los Angeles, CA 90095 USA; 8 Physics Department, Tianjin University, 300072 Tianjin, P. R. China

**Keywords:** Colloids, Liquid crystals, Self-assembly, Condensed-matter physics

## Abstract

Considering multi-body systems of monodisperse hard Brownian particles, it remains challenging to predict the forms of order that can emerge in their dense assembled structures. Surprisingly, here, using Monte Carlo simulations, we show that tetratic-ordered phases emerge in a dense two-dimensional system of hard kites that are rotationally asymmetric and have opposite 72° and *α* ≈ 90° internal angles. We observe a new tetragonal rectangular crystal (TRX) phase possessing (quasi-)long-range fourfold molecular-orientational order. We propose a method based on local polymorphic configurations of neighboring particle pairs (LPC-NPPs) to understand this emergent tetratic order and show that LPC-NPPs can be useful for predicting orientational order in such systems. To examine the dependence of the tetratic order on *α*, we apply LPC-NPP analysis to other hard kites for 54° ≤ *α* ≤ 144°. Our work provides insight into the creation of novel ordered materials by rationally designing particle shape based on anticipated LPC-NPPs.

## Abstract (web summary)

For colloidal particles, it remains challenging to predict the forms of order that can emerge in their dense assembled structures. Here, the authors show, by Monte Carlo simulations, that tetratic-ordered phases emerge in a dense two-dimensional system of hard kites that are rotationally asymmetric.

## Introduction

Achieving desired phases and structures by assembling shape-designed constituent particles is a promising yet quite challenging route for fabricating new functional materials^1^. Toward this goal, the physics governing the relationship between particle shape and assembled structures^1–3^, which could enable us to predict the forms of order of structures assembled by constitute monodisperse particles, still remains incomplete and therefore is worthy of highly detailed investigations. Two-dimensional (2D) systems of hard colloidal particles interacting with excluded volume interactions have long been used as model systems for such studies, because the phase behavior of such systems is solely determined by particle shape and their entropy. For example, for particles having a shape as simple as disk, their 2D system can show the hexagonal crystal phase, hexatic phase, and isotropic phase, and their melting transition is a two-step process via the hexatic phase^4–8^, which is different from the first-order melting transition in the three-dimensional (3D) system^9^. Compared with spherical particles, non-spherical colloidal particles like ellipses^10,11^ and rods^12–14^ can show more phases including nematic and smectic.

The tetratic phase is a type of liquid crystal phase which has fourfold rotational symmetry in molecular orientation. It is different from the biaxial nematic phase observed in a fluid composed of boomerang-shaped molecules^15,16^. In a tetratic phase, mesogens are orientated along two perpendicular directors. Whereas in a biaxial nematic phase, the orientation of mesogens is uniaxial, but mesogen’s long and short transverse axes are aligned along two perpendicular directors, respectively. The tetratic phase has been observed in simulations of hard squares^17^ as well as hard rectangles^13^. Experimentally, tetratic order has also been found in a system of colloidal rectangles^14^ and in a granular system of squares^18^. Both square shape and rectangle shape are intrinsically biaxial and have at least twofold rotational symmetry, which might facilitate the formation of tetratic order. This is supported by recent work on regular polygons whose results show the important role of rotational symmetry of polygons in their phase behavior^19–28^. For example, for regular hexagons^24^, a hexatic phase, a hexagonal rotator crystal, a hexagonal crystal, and a frustrated hexagonal crystal are found whereas for regular pentagons, whose fivefold rotational symmetry is not compatible with the symmetry of crystal structures, hexagonal rotator crystals and glass states are found in experiments under compression^21^. For plastic crystal phases, like the hexagonal rotator crystal, which have (quasi)long-range positional order and short-range orientational order, Shen et al.^26^ showed that their appearance in systems of regular polygons is dependent on the compatibility between the symmetry group of the particle shape and that of the local environment in the crystal. In addition, besides the rotational symmetry of particles, the tetratic phase seems also to be sensitive to the details of particle shape. Martínez-Ratón et al.^29^ have shown that a tetratic phase can be found in hard rectangles but not in hard discorectangles. Also, both experiments and simulations have shown that rounded squares have a quite different phase behavior than mathematically ideal squares, and no tetratic phase is observed in rounded squares^17,20,22,23^. However, it remains to be determined whether or not a single right internal angle in a convex polygonal shape, which is rotationally asymmetric, is enough to induce tetratic order in a slowly crowded Brownian system composed of many identical copies of that particular shape.

In materials other than liquid crystals, such as crystal phases, constituent particle orientations are also important for controlling their properties, including optical properties^30,31^. For instance, crystals containing particles with multiple orientations have been observed in 3D systems^32–34^. Alternatively, by using 2D periodic substrates, which create potential substrate minima that can trap colloids, novel colloidal molecular crystals are obtained. Multimers consisting of trapped multiple-charged colloids can form different orientational orders, such as an antiferromagnetic-like phase in which dimers are located on a square 2D periodic substrate but with perpendicular orientations between neighboring dimers^35–37^. In different 2D systems consisting of hard polygons, Shen et al.^26^ also reported a discrete plastic crystal of 8, 9, 10-gons in which the distribution of particle orientations have multiple peaks, which indicate that hard polygons can be possible candidates for assembling colloidal molecular crystals. But such discrete plastic crystals only show short-range order in molecular-orientation. Thus far, 2D discrete plastic crystals possessing (quasi)long-range molecular-orientational order have not been assembled by slowly crowding hard rotationally anisotropic particles; all prior examples all involve particle shapes that are at least twofold rotationally symmetric. In addition, a general method that can be used to predict the forms of self-ordering in slowly crowded Brownian systems of hard anisotropic particles of arbitrary shape is still lacking. Particularly, considering tetratic order, there has been no prior example of tetratically ordered phases, either crystalline or liquid crystalline, based on a particle shape that does not possess at least some form of rotational symmetry.

In this work, to address the above questions, by Monte Carlo (MC) simulations, we systematically studied an important class of shapes in two dimensions: hard kites. Our results show that tetratic order possessing fourfold rotational symmetry can be formed by particles that are completely rotationally asymmetric. A new crystal phase, tetragonal rectangular crystal (TRX) phase, which has (quasi-)long-range positional order and (quasi-)long-range fourfold molecular-orientational order, is observed. We propose a LPC-NPPs method to understand this emergent tetratic order and show that LPC-NPPs can be useful for predicting orientational order in such systems.

## Results

### Construction of kites

**Fig. 1:**
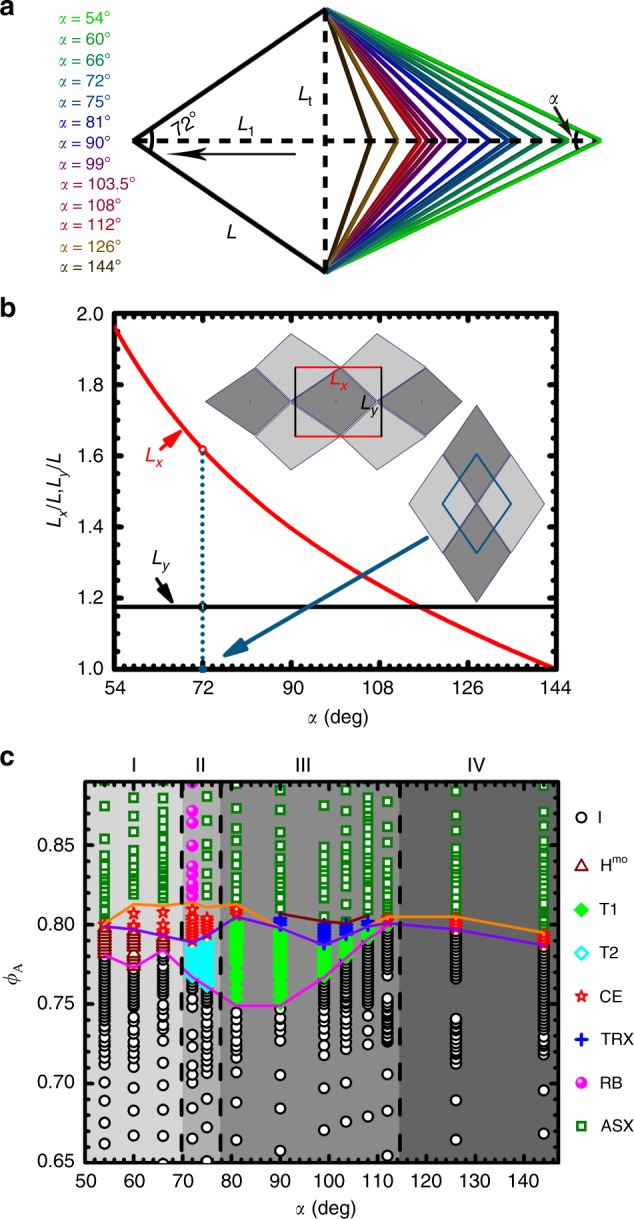
Sketch of kites and their phase diagrams obtained by MC simulations. **a** Sketch of kites with a fixed vertex angle of 72° and associated edge length of *L*, and a tunable shape parameter of angle *α* from 54° to 144°, whose vertex is opposite to the fixed 72° vertex. *L*_l_ is the length of particle diagonal connecting the vertices of 72° and *α*, *L*_t_ is the length of particle diagonal perpendicular to *L*_l_. *L*_l_/*L*_t_ is defined as the aspect ratio of particle. Black arrow in particle indicates the pointing direction from *α* vertex to the fixed 72° vertex, which is defined as the orientation of particle. **b** The length of two lattice vectors *L*_*x*_ and *L*_*y*_ of a unit cell when kites are in the closest packed ASX phase at different *α*. **c** Phase diagram of kites obtained from MC simulations using NPT and NVT ensembles. I: isotropic, H^mo^: hexatic phase in molecular-orientational order, T: tetratic, CE: coexistence, TRX: tetragonal rectangular crystal, RB: rhombic crystal, ASX: alternating striped crystal. Solid lines indicate the determined phase boundaries: dark purple line: phase boundaries between ASX and TRX; orange line: melting transition from crystal phase (ASX/RB or TRX) to liquid crystal phases or isotropic liquid phases; purple line: freezing transition from liquid crystal phases or isotropic liquid phases to crystal phases; magenta line indicates the phase boundaries between isotropic liquid phases and liquid crystal phases (tetratic or hexatic).

The kites are generated from a Penrose-kite shape by fixing three vertices that have an internal angle of 72° in the Penrose-kite, while moving the fourth vertex which has an internal angle of 144° in the Penrose-kite along its symmetry axis. For simplicity, we call the internal angle of the fourth vertex *α* (Fig. 1a). And the vertex opposite to the *α* vertex will have a fixed internal angle of 72° in all kites tested in this study. The orientation of a kite is defined as the pointing direction from *α* vertex to the fixed 72° vertex. The length of kites associated with the fixed 72° vertex is *L*. The aspect ratio of kites is defined as *L*_l_/*L*_t_, here *L*_l_ is the length of particle diagonal connecting the vertices of 72° and *α*, and *L*_t_ is the length of particle diagonal perpendicular to *L*_l_. In this study, we chose *α* to be 54°, 60°, 66°, 72°, 75°, 81°, 90°, 99°, 103.5°, 108°, 112°, 126°, and 144° (Fig. 1). When *α* = 72°, the particle shape becomes rhombus^38^, and when *α* = 144° the particle is Penrose-kite used in earlier works^28,39^. Except for the rhombus of *α* = 72°, all other kites tested in this study have fore-aft asymmetry and no rotational symmetry.

All particles designed in this study can fully tile the space with an alternating striped crystal (ASX) which has a complex rectangular lattice with a conventional unit cell containing two lattice points (see one example shown in Fig. 1b, where the conventional unit cell has lattice constants *L*_*x*_ = *L*_l_ and *L*_*y*_ = *L*_t_) except the case of *α* = 72°, where the shape of particles become rhombus and the ASX phase becomes rhombic crystal (RB) whose conventional unit cell is a simple rhombic cell (Fig. 1b). Previous work on Penrose kites has shown that the system can be easily frozen into a glassy state when it is under compression^28,40^. Since here we focus on the phase behavior in thermal equilibrium, we perform MC simulations in both NPT and NVT ensembles by performing an expansion starting from the densest packing structures (ASX) and studying the melting of those structures (see more details such as the equation of states, order parameters and correlation functions, etc. in Methods and Supplementary Methods, Supplementary Figs. 1–29).

### TRX and tetratic phases observed in kites

We first studied a set of hard (144°–*α*/2)–72°–(144°–*α*/2)–*α* kites (*α* from 90° to 108°) that contain at least a ~90° internal angle. Figure 1c shows the corresponding melting phase diagram (see more details in Supplementary Figs. 14–23). Besides the ASX phase and the isotropic liquid phase (I), surprisingly, two phases possessing tetratic order are observed.

**Fig. 2:**
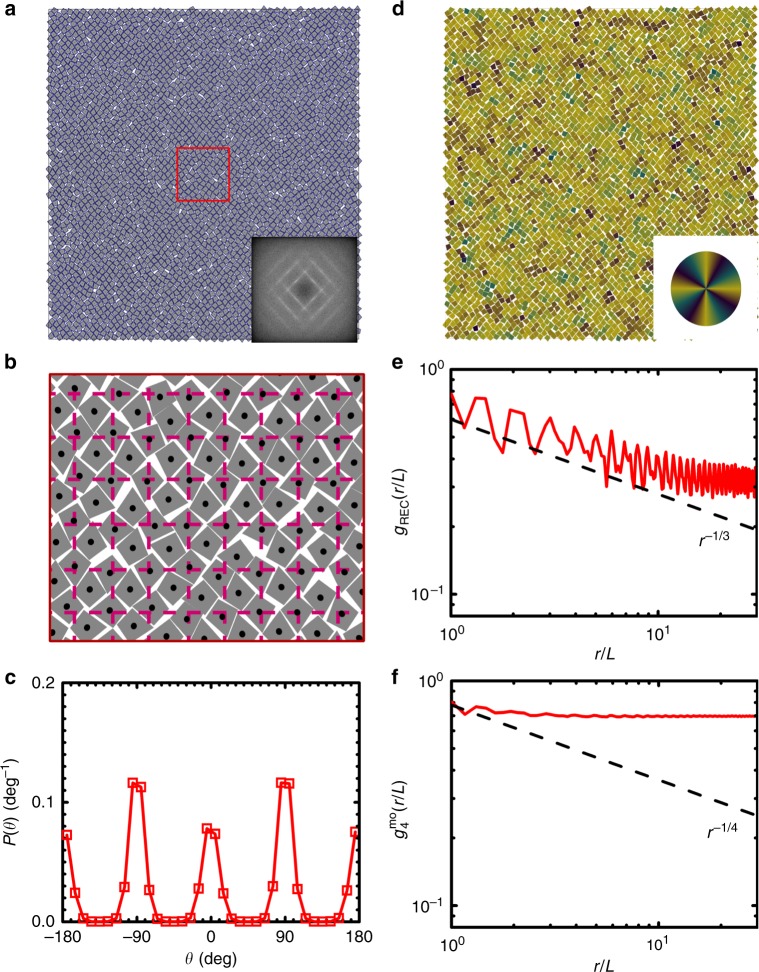
An example of a TRX phase (*α*  = 99°, *ϕ*_A_  = 0.800, NVT ensembles). **a** A snapshot of a configuration of kites. Inset shows the fast Fourier Transform (FFT) of the snapshot. **b** A magnified view of the enclosed region by the red square in **a**. Dashed lines show the rectangular lattice, which has lattice constants of 1.45*L* and 1.33*L*, obtained by maximizing the positional order parameter of the system. **c** The probability density *P*(*θ*) of the single particle pointing direction *θ*. **d** Configuration in **a** is color-coded by particles’ pointing directions. Inset shows the color wheel (with fourfold rotational symmetry) used for color-coding particle orientations. **e** The spatial correlation function of rectangular crystal lattice *g*_REC_(*r*). Dashed line is ∝ *r*^*−*1/3^, which is the KTHNY prediction for spatial correlation function at the crystal–liquid crystal transition point. **f** The fourfold molecular-orientational correlation function *g*_4_^mo^(*r*). Dashed line is ∝ *r*^−1/4^, which is the KTHNY prediction for the molecular-orientational correlation function at the liquid crystal-isotropic phase transition point.

One is a new crystal phase; it appears when systems melt from the ASX to the tetratic liquid crystal phase, which we call the TRX phase. The TRX phase keeps the symmetry of rectangular lattice in translation but has changed to a fourfold rotational symmetry in particle orientation as systems melt from the ASX phase. Figure 2 shows a representative configuration of particles in the TRX phase observed in kites of *α* = 99° at *ϕ*_A_ = 0.800, which has a global positional order parameter *S*_REC_ = 0.579 and a global fourfold molecular-orientational order parameter *ϕ*_4_ = 0.834. In the TRX phase, the centers of particles still approximately sit on a complex rectangular lattice (Fig. 2a, b), inherited from the ASX structure. However, the distribution of particle’s orientation shows four peaks in [−180°, 180°) which are separated by 90° (Fig. 2c). This indicates that particles in the TRX phase are not aligned along one axis (like particles in the ASX phase) but along two perpendicular axes. Graphically, this can be shown in a color-coded configuration in which particles are colored based on their orientations using a color wheel with fourfold rotational symmetry. And the result shows a homogeneous-colored graph (Fig. 2d). Quantitatively both the spatial correlation function of rectangular crystal lattice *g*_REC_(*r*) (Fig. 2e) and the fourfold molecular-orientational correlation function *g*_4_^mo^(*r*) (Fig. 2f) show a power law decay with exponents bigger than −1/3 and −1/4, respectively, indicating that the TRX phase has (quasi-)long-range order both in translation and in fourfold molecular orientation and is stable against the Kosterlitz–Thouless-type transition. This TRX phase is different from plastic crystal phases such as hexagonal rotator crystals reported in hard pentagons^21,27^, rounded squares^20,22,23^, and rounded hexagons^24^, in which particles are positioned on a hexagonal lattice but can rotate freely (thus no broken rotational symmetry).

**Fig. 3:**
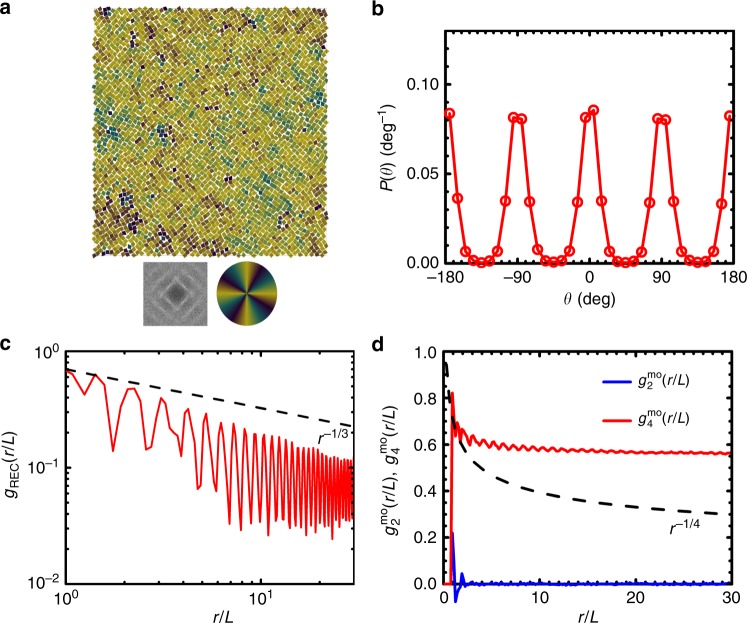
An example of a T1 phase (*α* = 90°, *ϕ*_A_ = 0.774, NPT ensembles). **a** A color-coded configuration using a color wheel with fourfold rotational symmetry. Bottom left: the fast Fourier Transform (FFT); bottom right: color wheel used for color-coding particle orientations. **b** Probability density *P*(*θ*) of single-particle orientational angle *θ*. **c** The spatial correlation function of rectangular crystal lattice *g*_REC_(*r*). Dashed line is ∝ *r*^−1/3^. **d** Two- and fourfold molecular-orientational correlation functions: *g*_2_^mo^(*r/L*) and *g*_4_^mo^(*r*/*L*). Dashed line is ∝ *r*^−1/4^.

The other phase having tetratic order is the tetratic liquid crystal phase (T1), observed at lower *ϕ*_A_. Figure 3a shows a color-coded configuration of particles using a color wheel with fourfold rotational symmetry in a typical tetratic phase obtained in kites of *α* = 90° at *ϕ*_A_ = 0.774. The homogeneity of color in it is consistent with a measured high value of fourfold molecular-orientational order *ϕ*_4_ = 0.753. Quantitatively, the probability density of particle orientation *P*(*θ*) shows four peaks with nearly equal peak values in [−180°, 180°) which are separated by 90° (Fig. 3b), indicating that in T1 phase particles have equal probabilities to align along two perpendicular axes (i.e., there is no preference between the two perpendicular axes for particles to align with). The tetratic phase has a low value of global positional order *S*_REC_ = 0.299 and does not have a stable quasi-long-range translational order (Fig. 3c). The measured twofold molecular-orientational correlation function *g*_2_^mo^(*r*) decays quickly to a near-zero level, while the fourfold molecular-orientational correlation function *g*_4_^mo^(*r*) decays algebraically and reaches to non-zero plateau which is much higher than the background level in the tested range of *r* (Fig. 3d), indicating a (quasi-)long-range fourfold molecular-orientational order in the tetratic phase.

### LPC-NPPs for understanding the emergent tetratic order

The kites of *α* = 90°, 99°, 103.5°, and 108° have no rotational symmetry but can form tetratic-ordered structures. Apparently, there is no direct correspondence between the symmetry of particle shape and the rotational symmetry of their assembled thermodynamic structures. What else would be critical properties of particles having different shapes in governing the rotational symmetry of structures that they thermodynamically assemble? In Penrose-kite systems (*α* = 144°), it has been shown that the local polymorphism of particles play a very important role in determining the structures of the condensed phase^28^. So in order to address the above question, here we have generalized this local polymorphism method and examined the local polymorphic configurations of neighboring particle pairs (LPC-NPPs) for kites having more general *α*.

**Fig. 4:**
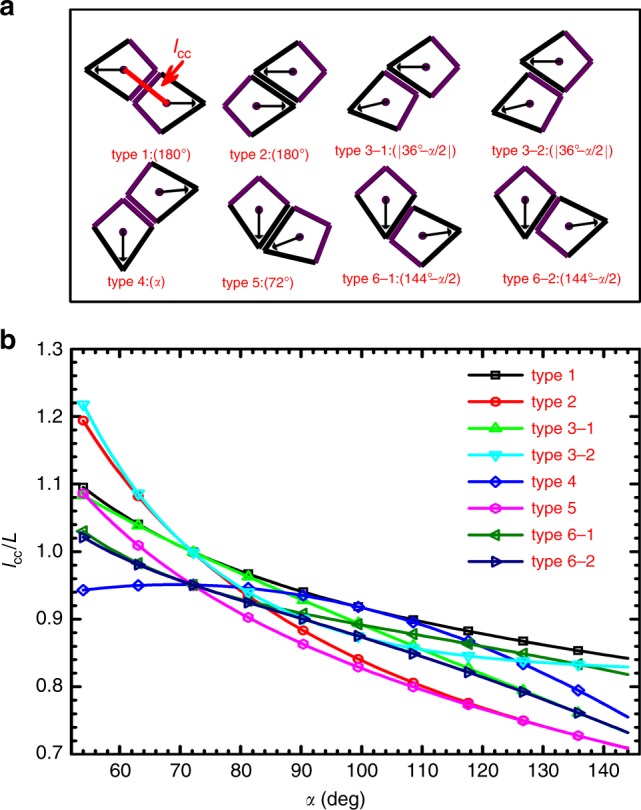
LPC-NPPs model. **a** Schematic graphs showing six types of LPC-NPPs. Numbers/expressions in parentheses show corresponding relative pointing angles *ϑ*. Note that for type 3 and type 6, one particle can slide relative to the other along the contacting edges while keeping the contact length maximized. In the graph, two cases for type 3 and type 6 are illustrated, corresponding to when the 72° vertex and the *α* vertex of one particle coincide with one of the non-72° and non-*α* vertices of the other particle, respectively. **b** Dimensionless center-to-center distance *l*_cc_/*L* of LPC-NPPs shown in **a** for kites with different *α*.

We first classify the possible LPC-NPPs of a kite shape into six types. As shown in Fig. 4a, in type 1 and type 4, the contacting edges are the edges with *α*-dependent lengths, whereas in type 2 and type 5, the contacting edges are the edges with fixed length *L*. In type 3 and type 6, one of the contacting edges has length *L*, and the other has an *α*-dependent length. Since the contacting edges in this case are not matched for kites of *α* ≠ 72°, one particle can slide relative to the other along the contacting edges while keeping the contacting part maximized. For example, type 3-1 and type 3-2 are two configurations when one particle slides relative to the other so that the 72° vertex and the *α* vertex of one particle coincides with one of the non-72° and non-*α* vertices of the other particle, respectively. Similar for type 6-1 and type 6-2. Figure 4b shows the calculated center–center distance *l*_cc_ of each type of LPC-NPPs from Fig. 4a.

Similar to the way used for Penrose kites, here each type of LPC-NPPs can be treated as an assembling unit (this is a reasonable assumption at high densities), then the appearance probability of each type of LPC-NPPs and their associated rotational symmetry properties will play important roles in determining the global rotational symmetry of the final assembly of those LPC-NPPs.

**Fig. 5:**
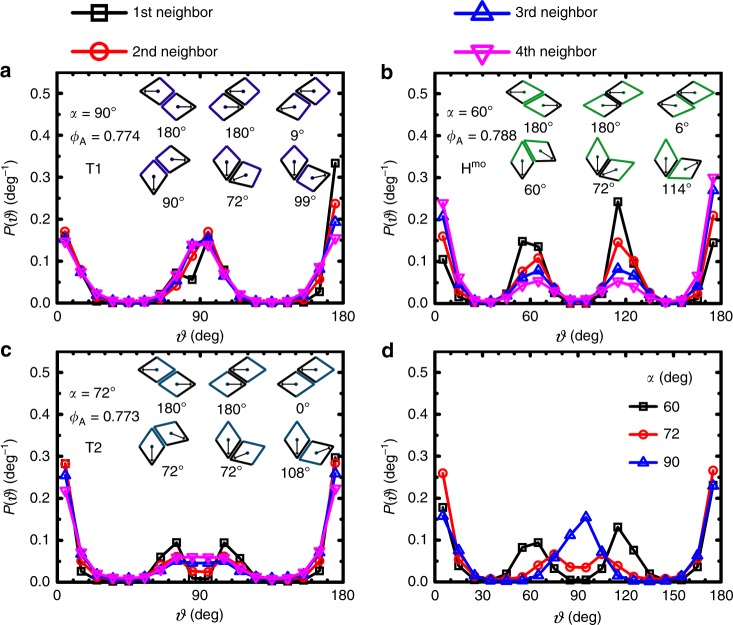
Relative pointing angle distribution *P*(*ϑ*). **a** *P*(*ϑ*) in a tetratic phase T1. **b** *P*(*ϑ*) in a hexatic phase. **c** *P*(*ϑ*) in a tetratic phase T2. The distributions of the first, second, third, and fourth nearest neighbors are shown. Insets illustrate LPC-NPPs with associated relative pointing angles for each type of kite. **d** *P*(*ϑ*) of the total four nearest neighbors for the three phases represented in **a**–**c**, clearly showing differences among the three phases.

To test this hypothesis, we then measured the distribution *P*(*ϑ*) of the relative pointing angle *ϑ* of neighboring particles (up to the first four nearest neighbors). Figure 5a shows *P*(*ϑ*)s between a center particle and its first, second, third, and fourth nearest neighbors in a representative tetratic phase formed by kites of *α* = 90° (see more results of other kites in Supplementary Fig. 30). Insets show the LPC-NPPs of kites. We can see that *P*(*ϑ*) of the first nearest neighbor shows peaks centered at 5°, 75°, 95°, and 175°, which match well with the relative pointing angles of its ideal LPC-NPPs type 3 (*ϑ* = 9°), type 5 (*ϑ* = 72°), type 6 (*ϑ* = 99°), and types 1 and 2 (*ϑ* = 180°), respectively, i.e., *P*(*ϑ*) of the first nearest neighbor is largely determined by the configurations of particles when they are in fully edge-edge contact. By contrast, *P*(*ϑ*)s of second, third, and fourth nearest neighbors show three peaks centered at 5°, ~90°, and 175°, which are broader and shallower compared with those of the first nearest neighbor. This is understandable, as neighboring particles with larger separation will have more room to move relatively and result in a broad range of relative pointing angles. Then the rotational symmetry of the final assembled structures will be largely determined by the sum of *ϑ* distributions from all neighbors including the first, second, third, and fourth nearest neighbors. And depending on the relative contribution between the first and the rest of nearest neighbors, the final *P*(*ϑ*) could be different from the one solely determined by the shape of particles. Taking kites of *α* = 90° (Fig. 5a) as an example, *P*(*ϑ*)s of second, third, and fourth nearest neighbors are clearly different from the one of the first nearest neighbor. Consequently, the total *P*(*ϑ*) shows three peaks around 0°, 90°, and 180° (Fig. 5d), which are all compatible with fourfold rotational symmetry and thus exhibit tetratic order.

### Application of LPC-NPPs in other kites of different *α*

To test the generality of LPC-NPPs method, we have applied it to understand the phase behavior of other kites with varying *α* (from 54° to 144°) (Fig. 1c) that we have simulated. For example, in systems of *α* =  60° (Fig. 5b), at *ϕ*_A_ = 0.788, *P*(*ϑ*) of the first nearest neighbor has peaks centered at 5°, 55°, 115°, and 175°, which correspond to the LPC-NPPs type 3 (*ϑ* = 6°), type 4 (*ϑ* = 60°), type 6 (*ϑ* = 114°), and types 1 and 2 (*ϑ* = 180°), respectively. While *P*(*ϑ*)s of second, third, and fourth nearest neighbors show four similar peaks except that peaks are broader and shallower compared with that of the first nearest neighbor. Then the total *P*(*ϑ*) has peaks centered around 0°, 60°, 120°, and 180° (Fig. 5d). This distribution is compatible with sixfold rotational symmetry and thus we would expect the system to have a hexatic order at the tested *ϕ*_A_. This is confirmed by the results obtained from the analysis of order parameters and correlation functions, which show that kites of *α* = 60° at *ϕ*_A_ = 0.788 are in a H^mo^ phase which has (quasi-)long-range sixfold molecular-orientational order but short-range positional order (Supplementary Figs. 4 and 5). Here, H^mo^ is used to differentiate it from the traditional hexatic phase (H) which has (quasi-)long-range sixfold bond-orientational order and short-range positional order^5^. Kites of *α* = 54° and 66° behave similarly (Supplementary Fig. 30a, b). But when *α* changes from 60° to 66° the peak values of *P*(*ϑ*) around 60°and 120° become smaller and thus have less prominent contributions to the final H^mo^ phase. This corresponds to a shorter existence window of H^mo^ phase observed in the kite of *α* = 66° than that in the kite of *α* = 60° (Fig. 1c).

Besides being found for kites of *α* = 90°, 99°, 103.5°, and 108°, the tetratic phase T1 is also observed in kites of *α* = 81°and 112°. But the relative contribution of each peak in *P*(*ϑ*) to the formed tetratic order varies for kites with different *α* (Supplementary Fig. 30d, h). In the tetratic phase of kites of *α*  = 81°, the peak values of *P*(*ϑ*) around 0° and 180° are both larger than the one around 90°; whereas for kites of *α* = 90°, the three peaks around 0°, 90°, and 180° have roughly equal values. As *α* continues to increase, the peak values around both 0° and 90° are gradually reduced. In the tetratic phase of kites of *α* = 112°, the peak around 0° is very small while the peak around 180° is much stronger than peaks at both 0° and 90°, indicating that most of neighboring pairs are anti-parallel, a remnant property from the ASX crystal. Following this trend, we would expect that for kites with higher *α*, when peaks around 0° and 90° becomes insignificant enough there will be no tetratic phase. This is consistent with our observations that there is no tetratic phase observed for kites of *α* = 126° and 144°.

**Fig. 6:**
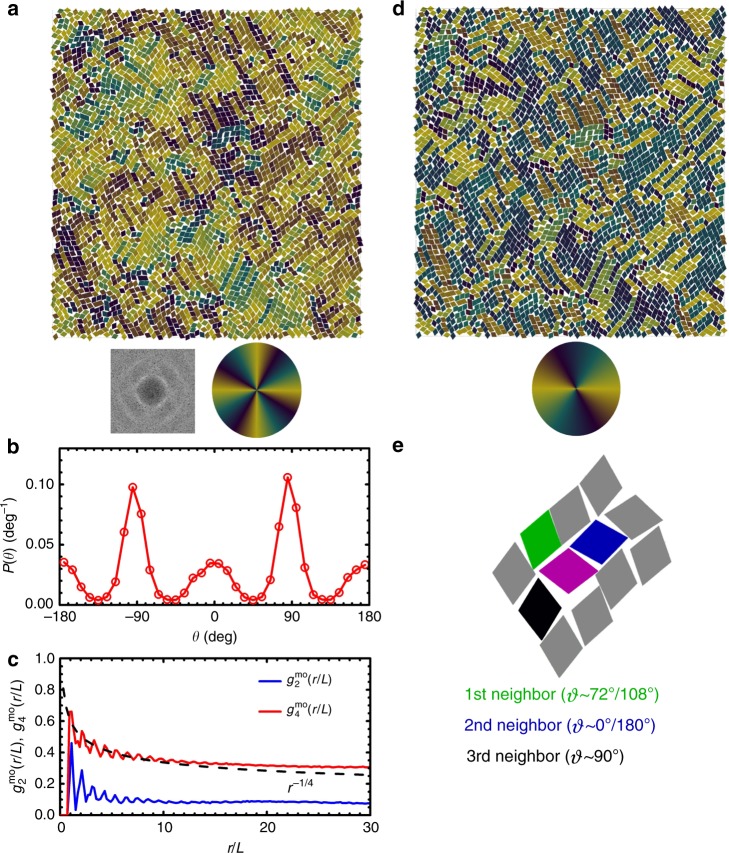
An example of a T2 phase (*α* = 72°, *ϕ*_A_ = 0.773, NPT ensembles). **a** A color-coded configuration using a color wheel with fourfold rotational symmetry. Bottom left: the fast Fourier Transform (FFT); Bottom right: color wheel used for color-coding particle orientations. **b** Probability density *P*(*θ*) of single-particle orientational angle *θ*. **c** Two- and fourfold molecular-orientational correlation functions *g*_2_^mo^(*r/L*) and *g*_4_^mo^(*r/L*). Dashed line is ∝ *r*^−1/4^. **d** The same configuration as in **a** but color-coded using a color wheel with twofold rotational symmetry. Bottom: color wheel used for color-coding particle orientations. **e** A representative configuration of twinning structure showing *ϑ* ~ 90° between the target particle (magenta color) and its third nearest neighbor (black color) in *α*  = 72° system. The emergent domain structures seen in parts **a** and **d** can be interpreted morphologically as twinning.

**Fig. 7:**
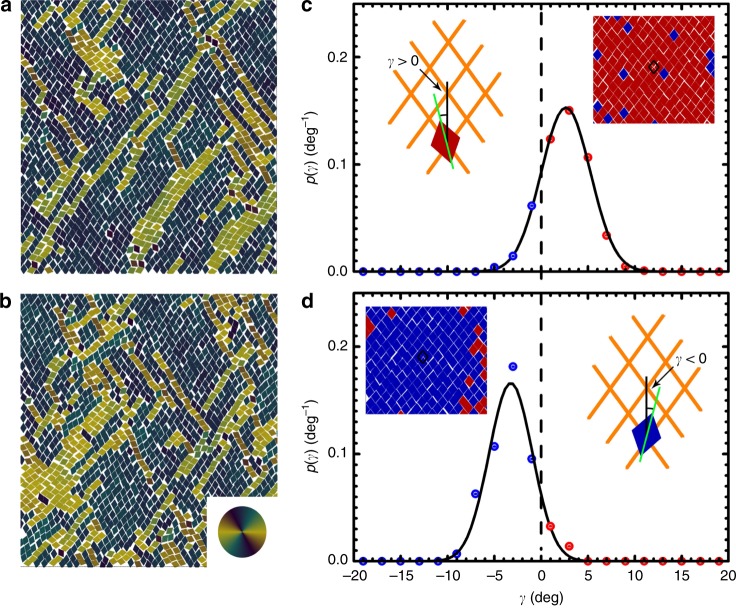
Twinning structures and the emergence of chirality in RB crystals. **a** Polysynthetic twin and **b** cyclic twin structures formed by kites of *α* = 72° at *ϕ*_A_ = 0.798 and 0.787, respectively, obtained by MC simulations (NPT ensemble). Particles are colored based on their orientations. Color wheel: encodes particle orientations with twofold symmetry. **c**, **d** Evidence of chiral symmetry breaking in RB crystals: tilting of rhombs in kites of *α* = 72° at *ϕ*_A_ = 0.906, obtained by MC simulations (NPT ensemble). Probability distribution, *P*(*γ*) (in deg^−1^), where *γ* is the angle difference between the orientation of a rhombus and one diagonal direction of the RB crystal lattice. If the orientation of the rhombus deviates clockwise from the diagonal lattice direction, then *γ* is negative (blue points and rhombs); otherwise, *γ* is positive (red points and rhombs). Black solid lines: least-squares fits to Gaussian functions. **c** Example of positive-deviation RB crystal. Left inset: schematic showing *γ* > 0 for a single rhomb. Right inset: color-coded micrograph that has *γ*_ave_ > 0; the black rhombic outline in the micrograph shows a unit cell of the RB lattice. **d** Example of negative-deviation RB crystal. Left inset: color-coded micrograph that has *γ*_ave_ < 0; the black rhombic outline in the micrograph shows a unit cell of the RB lattice. Right inset: schematic showing *γ* < 0 for a single rhomb.

Interestingly, kites of *α* = 72° (i.e. 72° rhombus) and 75° show a different tetratic phase T2. For a configuration of 72° rhombs at *ϕ*_A_ = 0.773 (Fig. 5c), *P*(*ϑ*)s of first, second, and third nearest neighbors are similar and show four peaks centered at 5°, 75°, 105°, and 175°. These are different from *P*(*ϑ*) of fourth nearest neighbors (kites of *α* = 75° show similar behavior (Supplementary Fig. 30c)). In this case, the total *P*(*ϑ*) shows two relatively sharp peaks at around 0° and 180°, and a broad plateau centered at 90° resulted from merging of two weak peaks centered at 72° and 108°, respectively (Fig. 5d). As a result, the system exhibits a global fourfold symmetry in molecular orientation, and is thus classified to be in a tetratic phase. But this tetratic phase (T2) has a clearly different microscopic structure than T1 observed in kites of *α* = 90° as illustrated in Fig. 3. Figure 6a shows one example of T2 phase observed for kites of *α* = 72° at *ϕ*_A_ = 0.773, which is color-coded using the same color wheel as in Fig. 3a. It shows a palette-like pattern consisting of patches with different colors, which is different from the homogeneous-colored pattern in the T1 phase (Fig. 3a). The peaks of *P*(*θ*) are much higher at *θ* = ±90° than at *θ* = ±180° and 0°, indicating that in T2, particles prefer to align along one axis than the other (Fig. 6b). This can also explain the observations that in T2, *g*_2_^mo^(*r*) does not decay to zero and the plateau value that *g*_4_^mo^(*r*) reaches at large distance is smaller than that in T1 (Figs. 6c and 3c). To further understand the local structures in T2, Fig. 6d shows the same configuration but color-coded using a color wheel with twofold rotational symmetry. It can be seen that in T2, particles form nematic domains and in each domain particles are aligned along a same axis, but the orientation of the axis of each domain varies. A close examination of nematic domains in T2 reveals that neighboring nematic domains typically form twinning structures (see one example in Fig. 6e). These observations are consistent with the measured *P*(*ϑ*) shown in Fig. 5d, which shows relatively strong peaks centered at 0° and 180°, presumably contributed by particles in the nematic domains, and shows two relatively weak peaks centered at 72° and 108°, contributed mostly by particles around twinning structures, i.e., by particles around boundaries between nematic domains. The two weak peaks merge with each other and result in an elevated *P*(*ϑ*) at 90°, which will contribute positively to the fourfold rotational order but negatively to the twofold rotational order of the system. This is similar to the tetratic order formed by the proliferation of grain boundaries which disrupt nematic order but preserve tetratic order during the transition from nematic to isotropic in colloidal rectangles^14^. We note that for kites of *α* = 72°, similar to the observed twin structures in experiments^38^, in the configurations obtained by MC simulations, we also found polysynthetic twin as well as cyclic twin structures during the expansion of RB phases as *ϕ*_A_ is lowered (Fig. 7a, b). Moreover, domains of single RB crystal, which exhibits emergent chirality, are also observed in simulations (Fig. 7c, d), which again agree with earlier experimental^38^ and simulation results^41^. However, the phase behavior of rhombs (i.e., kites of *α* = 72°) obtained from MC simulations shows a sequence of RB-T2-I as the system melts; this is different from the experimental observations for corner-rounded rhombs, where a phase sequence of I–H–hexagonal rotator crystal (RX)–RB is observed as this rhombus system is slowly compressed^38^. One possible reason that could potentially account for this difference is the corner roundness of rhombus used in experiments, as corner roundness of particles has been shown to affect the phase behavior dramatically in crowded systems of squares^17,20,22,23^. But to test this hypothesis, more work is needed.

## Discussion

Tetratic phase has been reported in the systems of hard rectangles with an aspect ratio between [1, 2.21]^13^, including the special case of squares which have an aspect ratio of 1 (refs. ^17,22^). The kites studied in this work have a range of aspect ratio of [0.85, 1.67] (Supplementary Fig. 31. Note that for kites of *α* = 126° and 144°, the aspect ratio which is defined by *L*_l_/*L*_t_, is <1 since for the two kites *L*_t_ > *L*_l_. They correspond to 1.06 for kite of *α* = 126° and 1.18 for kite of *α* = 144°, when the aspect ratio is defined by the ratio of the longer diagonal length divided by the shorter diagonal length). However, only kites of *α* between 72° and 112°, which have a range of aspect ratio of [1.03, 1.38], show a tetratic phase, indicating that compared with rectangles, the kites have a smaller existence window of tetratic phase in the range of aspect ratio.

TRX phase observed in this study has not been reported before. In this phase, centroids of kites still form a complex rectangular lattice while their orientations are aligned along two perpendicular axes. The TRX phase is observed in kites of *α* = 90°, 99°, 103.5°, and 108°. These four types of kites are more close to a disk than other tested kites, indicated by their higher values of isoperimetric quotient^25^ IQ = $4\pi A_{\mathrm{P}}/C_{\mathrm{P}}^2$, where *A*_P_ and *C*_P_ are the area and perimeter of particles, respectively (Supplementary Fig. 31). Moreover, from the Fig. 4b, we can see that for kites of *α* = 90°, 99°, 103.5°, and 108°, the *l*_cc_ of six LPC-NPPs can be roughly categorized into three groups, with each group having two types of LPC-NPPs that have approximately equal *l*_cc_. For example, for kite of *α* = 99°, the *l*_cc_ of type 1 is approximately equal to the one of type 4, similarly, type 2 and type 5, type 3 and type 6 also have equal *l*_cc_, respectively. Together, these results indicate that kites of *α* = 90°, 99°, 103.5°, and 108° would be easier to rotate and thus certain LPC-NPPs types can interchange without causing much variation in free space. In other words, taking kites of *α* = 99° as an example, type 1 can be replaced by type 4 without inducing a big change in the centroid positions of particles. Such change in types would have minor effect on the translational order, but would affect the orientational order very much because the relative pointing angle of each type varies. In the case of kites of *α* = 99°, when one LPC-NPP changes from type 1 to type 4, *ϑ* of the corresponding neighboring pair changes from 180° to 99°. Similarly, when one LPC-NPP changes from type 2 to type 5, *ϑ* of the corresponding neighboring pair changes from 180° to 72°, and so on, so forth. At the end, together with thermal diffusion of particles which will lead to a wider range of particle orientations, the final effect of these changes will result in a total distribution *P*(*ϑ*) showing three peaks around 0°, 90°, and 180°, a feature of global fourfold rotational symmetry. But the positional order can still be maintained as the spacing between neighboring particles does not change much and particles still approximately sit on a complex rectangular lattice sites. The TRX phase observed in this work is different from discrete plastic crystals of 8, 9, 10-gons reported by Shen et al.^26^. In the kite systems, the TRX phase is an intermediate phase between ASX phase and tetratic phase with (quasi-)long-range order in fourfold molecular orientation, while the discrete plastic crystal phase is an intermediate phase between crystal phase and hexatic fluid/isotropic fluid phase with short-range order in molecular orientation. In the future, it will be very interesting to see whether other values of the fixed angle of kites (i.e. other than 72°) can also lead to TRX phases.

In summary, by MC simulations we systematically investigated the melting phase behavior of a series of kites with different *α*, which can be roughly classified into four groups based on the type of liquid crystals that they can form (Fig. 1c). Group I includes kites of *α* = 54°, 60°, and 66°, which show an ASX-H^mo^-I melting sequence. There is a coexistence (CE) region between H^mo^ and ASX (Supplementary Fig. 32), indicating a first-order ASX-H^mo^ transition. Group II includes kites of *α* = 72° and 75°, which show T2 phase that has global tetratic but microscopically nematic-like structures, while group III includes kites of *α* from 81° and 112°, which show T1 phase in which particles form assembled structures with microscopically uniform tetratic order. Group IV includes kites of *α* = 126° and 144°, which show a direct melting from ASX to isotropic phase with a CE region in between (Supplementary Fig. 33). Finite system-size effects can influence the precise determination of phase boundaries and the nature of the associated phase transitions, and simulations on larger systems could add additional precision to the location and nature of the phase boundaries that we have reported.

Our results showed that tetratic order which has fourfold rotational symmetry can be formed by particles with no rotational symmetries. A new crystal phase, TRX phase which has (quasi-)long-range order in both position and fourfold molecular orientation, is observed for kites of *α* = 90°, 99°, 103.5°, and 108°. Together with the observation that the tetratic phase is found in kites of *α* from 72° to 112°, these results indicate that the right angle in a polygonal shape is not a necessary condition but will facilitate to show a global tetratic order of their assembled structures. We generalized the concept of local polymorphic configurations, developed initially to explain glassy behavior of Penrose kites, to propose a LPC-NPPs method, which can explain the observed four(six)fold rotational symmetry in the T(H^mo^) phase of kites whose shape have no rotational symmetry (or have twofold rotational symmetry for rhombus), indicating that the LPC-NPPs method can be useful as a tool for predicting orientational order in systems as one or more shape parameters are systematically varied. To develop a more complete model involving multiple particles based on the number of local particles and relative configurations, which are needed in order to predict behavior accurately without requiring full-scale simulations, is very interesting and certainly worth of further studies. Thus, to design the shape of particles for assembling, not just the shape itself, but the local polymorphic configurations of two or more particles also need to be taken into consideration. Our results provide insight into controlling the bottom-up assembly-based fabrication of new functional materials by rationally designing the shape of constituent particles and their associated LPC-NPPs.

## Methods

### MC simulations

We performed MC simulations in isobaric-isothermal NPT and canonical NVT ensembles^42^ to investigate the melting phase behavior of hard kites. Simulations are carried out in a square box with periodic boundary conditions. The number of simulated particles is in a range of [3168, 3876] (see Supplementary Table 1). In the NPT ensemble, perfect ASX crystal configurations (see Fig. 1b in the main text) are used as the initial states for expansion (melting) runs. In each MC step, there are *N* particle trial moves (each particle will have a trial move once) and two box trial moves. A trial move of a particle consists of both translation and rotation, and the amplitude of one-dimensional translational displacement is proportional to the amplitude of rotational displacement by a factor of $\sqrt {I/m}$ based on equal partition theorem, where *m* is the mass and *I* is the moment of inertia of particle. In both particle and box trial moves, the acceptance ratio is set to be 40%. The system first run 4.5 × 10^6^ steps to equilibrate, and then run 0.5 × 10^6^ steps for statistical ensemble average. In the NVT ensemble, we first choose a defect-free equilibrated structure with a slightly higher density than the target density obtained in the NPT process, and then expand it so that the resulted configuration has the target density, which serves as initial configuration. For simulations in the NVT ensemble, in each MC step, there are only *N* particle trial moves. Similar to the NPT ensemble, the system first run 4.0 × 10^6^ steps to equilibrate, and then run 1.0 × 10^6^ steps for statistical ensemble average. Reduced units are used in simulations and the reduced pressure and area fraction are defined as $P^ \ast = PL^2/k_{\mathrm{B}}T$ and *ϕ*_A_ = *NA*_p_/*A*, where *P*, *L*, *N*, *k*_B_, *A*_p_, and *A* are pressure, length of fixed edges of kite, total particle number, Boltzmann’s constant, particle area, and the total area of system, respectively. Supplementary Figure 1 shows the obtained phase diagram using NPT (Supplementary Fig. 1a) and NVT (Supplementary Fig. 1b) ensembles. An expanded view of the combined phase diagram between *ϕ*_A_ = 0.74 and *ϕ*_A_ = 0.82 is shown in Supplementary Fig. 1c.

To test the effect of the shape of boundary conditions on the observed phases, using kites of *α* = 99° as an example, we performed additional MC simulations using both an isothermal–isostress *Nσ*_P_*T* ensemble, which allows triclinic changes in the shape of the boundary box, and a NPT ensemble but with a 60° rhombic-shaped box (Supplementary Figs. 18 and 19). The results show the same phase sequences as observed in the NPT ensemble with a square box, indicating that the effect of the shape of boundary conditions on our observed structures is negligible.

### Order parameters and correlation functions

Phases of kites obtained from MC simulations are determined by order parameters and correlation functions. The global *n*-fold molecular-orientational order parameter is defined as

1 $$\phi _n = \frac{1}{N}\mathop {\sum}\limits_{i = 1}^N {{\mathrm{e}}^{ - {\mathrm{i}}n\theta _i}} ,$$

Where *θ*_*i*_ is the angle of orientation of particle *i*. To calculate the global *n*-fold bond-orientational order parameter *Ψ*_*n*_, the local *n*-fold bond-orientational order *φ*_*n*_ is first calculated as

2 $$\varphi _n\left( {{\mathbf{r}}_i} \right) = \frac{1}{{N_i}}\mathop {\sum}\limits_{k = 1}^{N_i} {{\mathrm{e}}^{ - {\mathrm{i}}n\theta _{ik}}} ,$$

where *N*_*i*_ is the number of nearest neighbors of particle *i*, and *θ*_*ik*_ is the angle between an arbitrary fixed axis and the line connecting the centers of particles *i* and *k*. For *n* = 4, the first four nearest neighbors are used; and for *n* = 5 and 6, the neighbors are obtained through Voronoi construction. Then *Ψ*_*n*_ is defined as

3 $$\varPsi _n{\mathrm{e}}^{{{i}}\omega } = \frac{1}{N}\mathop {\sum}\limits_{i = 1}^N {\varphi _n} \left( {{\mathbf{r}}_i} \right),$$

where *ω* represents a global phase. Similarly, the local positional order parameter *ζ* for each particle *i* is defined as

4 $$\zeta \left( {{\mathbf{r}}_i} \right) = {\mathrm{e}}^{ - {{i}}{\mathbf{G}} \cdot {\mathbf{r}}_i},$$

where ${\mathbf{G}}$ is the reciprocal lattice vector of appropriate crystal lattice. And the global positional order parameter *S* is defined as

5 $$S = \left| {\frac{1}{N}\mathop {\sum}\limits_{i = 1}^N {\zeta \left( {{\mathbf{r}}_i} \right)} } \right|.$$

In this study, the positional order parameters of square *S*_SQ,_ hexagonal *S*_HEX_, rectangular *S*_REC_, and rhombic *S*_RB_ crystals are calculated. Using standard conventions, the susceptibility of bond-orientational order parameter is defined as

6 $${{\chi }_n} = N ( {\langle {\varPsi }_{n}^2 \rangle }- {\langle {\varPsi }_{n}\rangle }^2 ),$$

where $N$ is the number of particles. The *n*-fold molecular-orientational correlation function is defined as

7 $$g_n^{{\mathrm{mo}}}\left( r \right) = \left\langle {{\mathrm{cos}}\left[ {n\theta \left( {{\mathbf{r}}_i + r} \right) - n\theta \left( {{\mathbf{r}}_i} \right)} \right]} \right\rangle ,$$

the *n*-fold bond-orientational correlation function is defined as

8 $$g_n\left( r \right) = {\mathrm{Re}}\left\langle {\varphi _n^ \ast \left( {{\mathbf{r}}_i} \right)\varphi _n\left( {{\mathbf{r}}_i + r} \right)} \right\rangle ,$$

and the spatial correlation function relating to positional order is defined to be

9 $$g_S\left( r \right) = {\mathrm{Re}}\left\langle {\zeta ^ \ast \left( {{\mathbf{r}}_i} \right)\zeta \left( {{\mathbf{r}}_i + r} \right)} \right\rangle .$$

Here, Re represents an operator returning the real part of the value, and < > means taking the ensemble average.

The phase boundaries are estimated by a combination of calculated order parameters, correlation functions, susceptibilities of bond-orientational order parameters, and analyzed configuration images.

## Supplementary information

Supplementary Information

## Data Availability

The data that support the findings of this study are available from the corresponding author upon reasonable request.

## Code availability

Code for MC simulations used in this study are available from the corresponding author upon reasonable request.
