# Supplementary material for: Emergent tetratic order in crowded systems of rotationally asymmetric hard kite particles
Source: Nat Commun. 2020 Apr 28;11:2064. doi: 10.1038/s41467-020-15723-w (PMC7188800; doi:10.1038/s41467-020-15723-w)
Supplement: Supplementary file 1 — Supplementary Information [file 41467_2020_15723_MOESM1_ESM.pdf]

Supplementary Information for

Emergent tetratic order in crowded systems of rotationally asymmetric hard kite particles

Hou et al.

Supplementary Table 1. The number of particles used in simulations

α (degree)	Number of particles	α (degree)	Number of particles
54	3224	99	3520
60	3536	103.5	3520
66	3570	108	3520
72	3456	112	3280
75	3876	126	3360
81	3478	144	3760
90	3168		

Supplementary Methods

In 2D *NPT* ensembles, surface area *A* is treated as an additional coordinate to allow systems to transform freely[1], and the statistical error of the surface area *A* and also the area fraction $\phi_A = NA_p/A$ is inversely proportional to $\sqrt{N}$. Here, *N* is the total number of particles. The simulated *NPT* systems employed in this study have *N* in a range of 3168 ~ 3876 (Supplementary Table 1), so for *NPT* ensembles, the relative error of ϕ_A is estimated to be $1/\sqrt{N}$ ~ 0.02. Such magnitude of relative error can hide certain phases if the stable existence window of ϕ_A for such phases is comparable to the statistical error of ϕ_A. To check the possible hidden phases due to the error in ϕ_A in *NPT* ensembles, we also performed MC simulations using 2D *NVT* ensembles (in 2D, *V* is replaced by *A*) in the vicinity of phase transition points.

Supplementary Figure 1 shows the obtained phase diagram using *NPT* (Supplementary Figure 1(a)) and *NVT* (Supplementary Figure 1(b)) ensembles. Generally, the phase behavior obtained by both *NPT* and *NVT* ensembles are consistent with each other, but there are some differences due to the fluctuation of ϕ_A in *NPT* systems (Supplementary Figure 1): firstly, there are phases that are observed in *NVT* systems but not in *NPT* systems, such as H^{mo} phase in kites of α = 54° and 66°, coexistence region in kites of α = 81°, 126° and 144°, TRX phase in kites of α = 90° and 108°, and tetratic phase in kites of α = 103.5°, 108° and 112°. Those phases observed in *NVT* systems show a relatively narrow existence window of area fraction (<0.02), which is comparable to the fluctuation of ϕ_A (~0.02) in *NPT* systems tested in this study. We expect that those phases would be observable in larger *NPT* systems. Secondly, the phase boundaries obtained in *NPT* and *NVT* systems exhibit slightly deviation due to the fluctuation of ϕ_A in *NPT* systems.

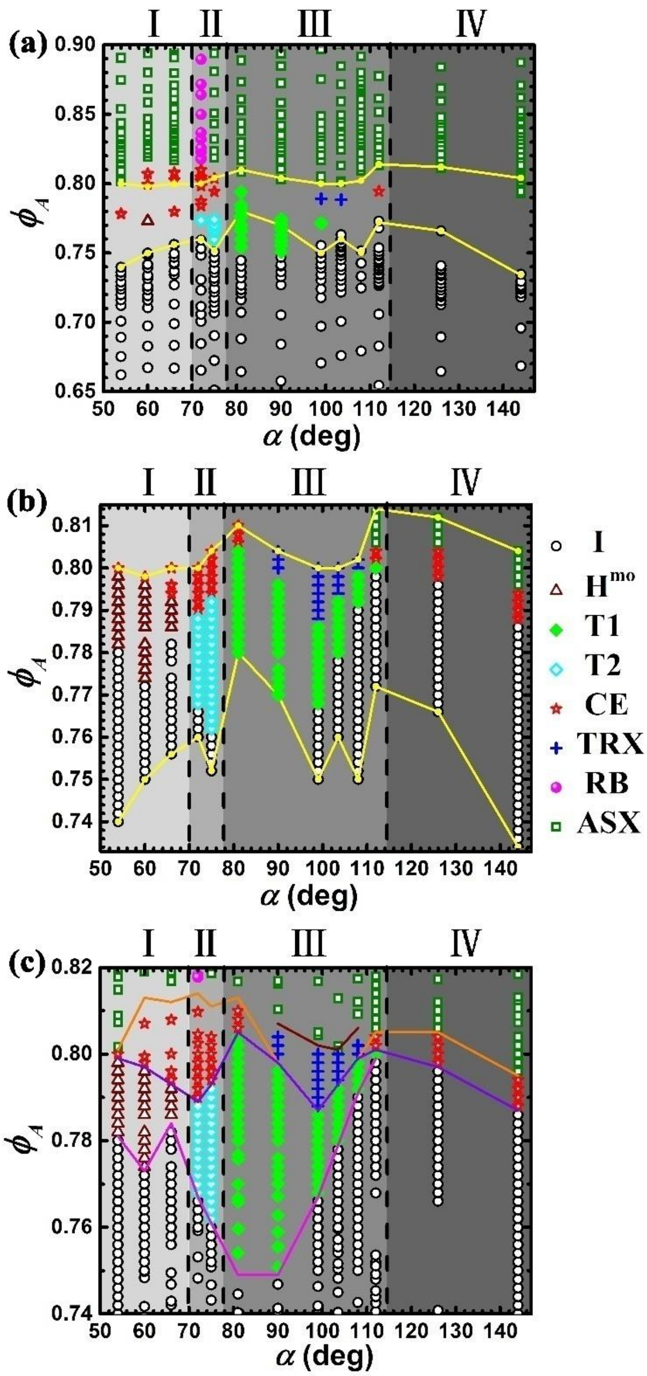

Supplementary Figure 1. Phase diagram of kites obtained from MC simulations. Results obtained using (a) *NPT* and (b) *NVT* ensembles. The *NVT* results only show phase diagrams at the ϕ_A near phase transitions (corresponding to the region between yellow lines in (a)). I (isotropic); H^{mo} (hexatic phase in molecular-orientational order); T (tetratic); CE (coexistence); TRX (tetragonal rectangular crystal); RB (rhombic crystal); ASX (alternating striped crystal). (c) An expanded view of Figure 1(c) between $\phi_A = 0.74$ and $\phi_A = 0.82$. Solid lines indicate the determined phase boundaries.

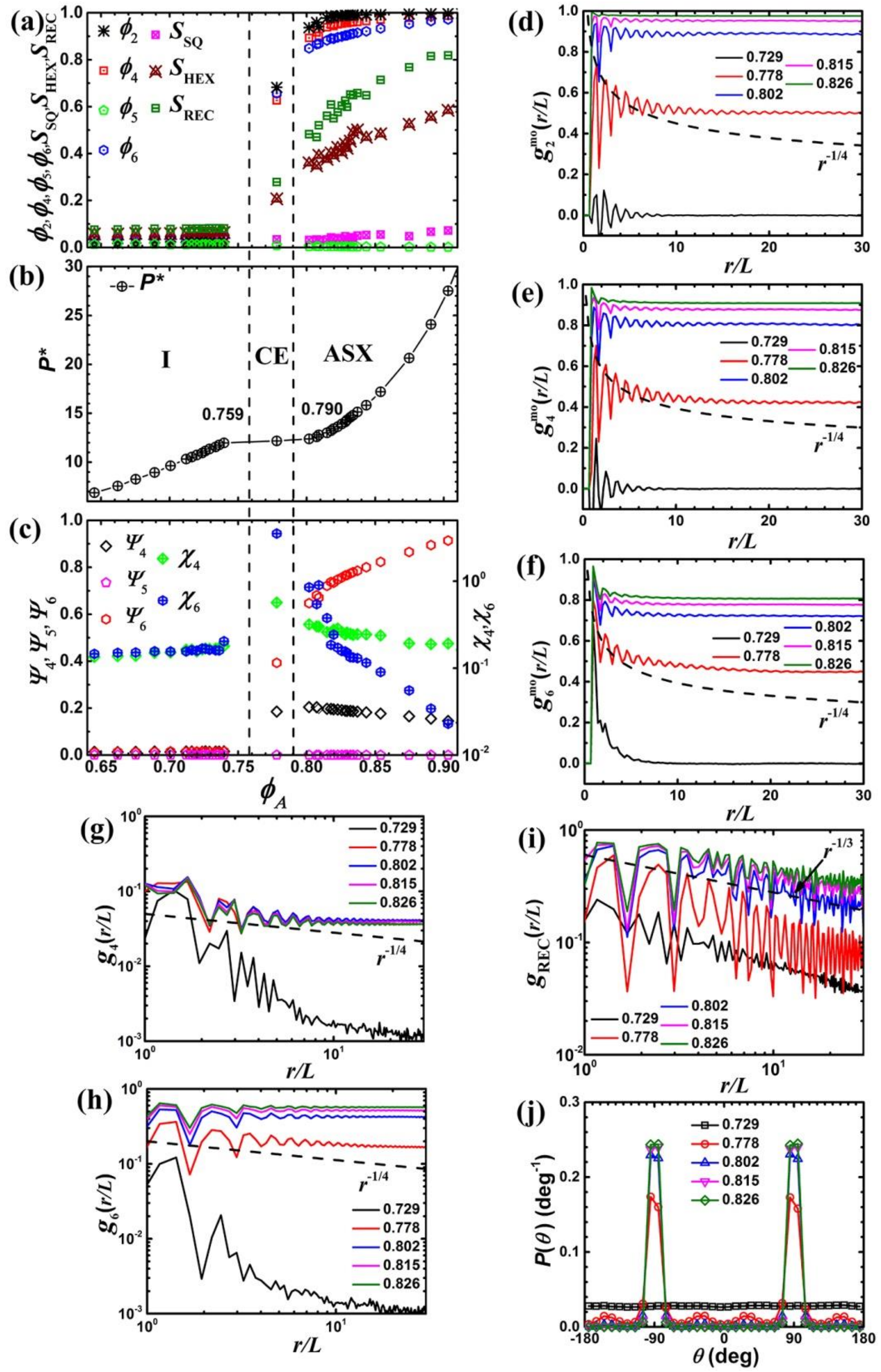

Supplementary Figure 2. Order parameters and correlation functions for different phases of kites of α = 54° obtained by *NPT* simulations. (a) Molecular-orientational order parameters, ϕ_2, ϕ_4, ϕ_5, and ϕ_6, positional order parameters of square lattice S_{SQ}, hexagonal lattice S_{HEX}, and rectangular lattice (complex lattice) S_{REC}; (b) Equation of state (EOS) with reduced pressure $P^* = PL^2/k_BT$; (c) Bond-orientational order parameters, Ψ_4, Ψ_5 and Ψ_6, and

susceptibilities of bond-orientational order parameters χ_4 and χ_6. Vertical dashed-lines are used to delimit different phases; (d-f) Molecular-orientational correlation functions, $g_2^{mo}(r)$, $g_4^{mo}(r)$ and $g_6^{mo}(r)$; (g-h) Bond-orientational correlation functions, $g_4(r)$ and $g_6(r)$; (i) Spatial correlation function of rectangular lattice, $g_{REC}(r)$; (j) Distribution $P(\theta)$ of single-particle orientation. Dashed lines in (d-i) are curves $\propto r^{-1/4}$ or $r^{-1/3}$.

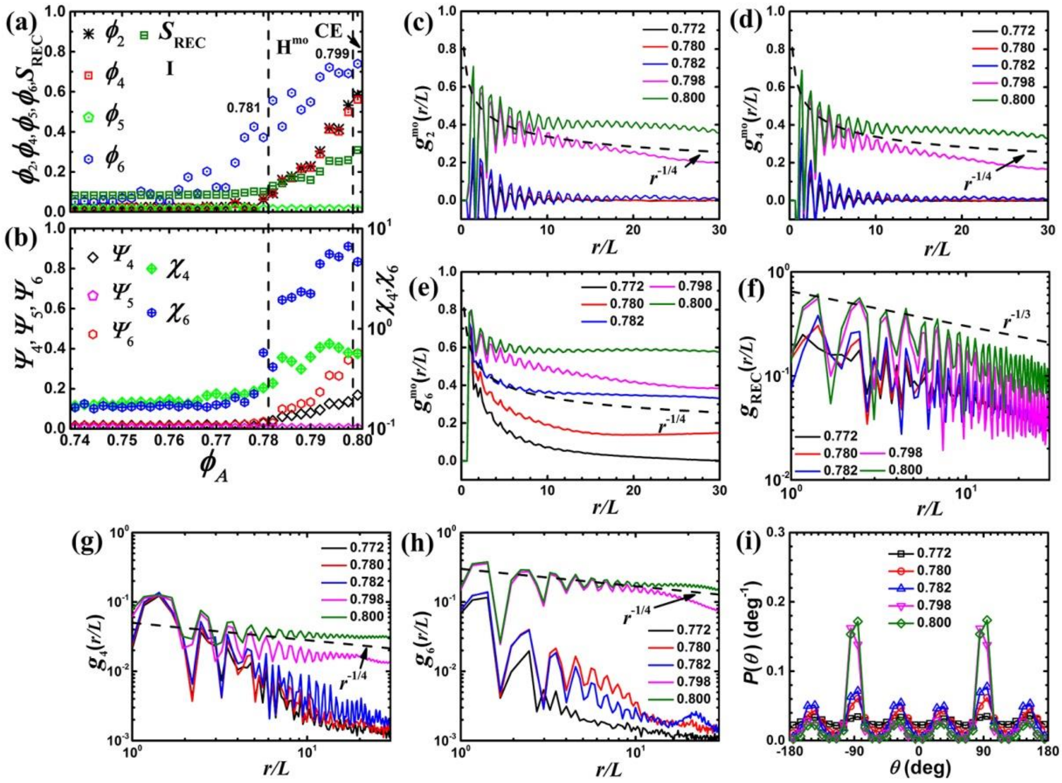

Supplementary Figure 3. Order parameters and correlation functions for different phases of kites of α = 54° obtained by *NVT* simulations. (a) Molecular-orientational order parameters, ϕ_2, ϕ_4, ϕ_5, and ϕ_6, positional order parameters of rectangular lattice (complex lattice) S_{REC}; (b) Bond-orientational order parameters, Ψ_4, Ψ_5 and Ψ_6, and susceptibilities of bond-orientational order parameters χ_4 and χ_6. Vertical dashed-lines are used to delimit different phases; (c-e) Molecular-orientational correlation functions, $g_2^{mo}(r)$, $g_4^{mo}(r)$ and $g_6^{mo}(r)$; (f) Spatial correlation functions of rectangular lattice, $g_{REC}(r)$; (g-h) Bond-orientational correlation functions, $g_4(r)$ and $g_6(r)$; (i) Distribution $P(\theta)$ of single-particle orientation. Dashed lines in (c-h) are curves $\propto r^{-1/4}$ or $r^{-1/3}$.

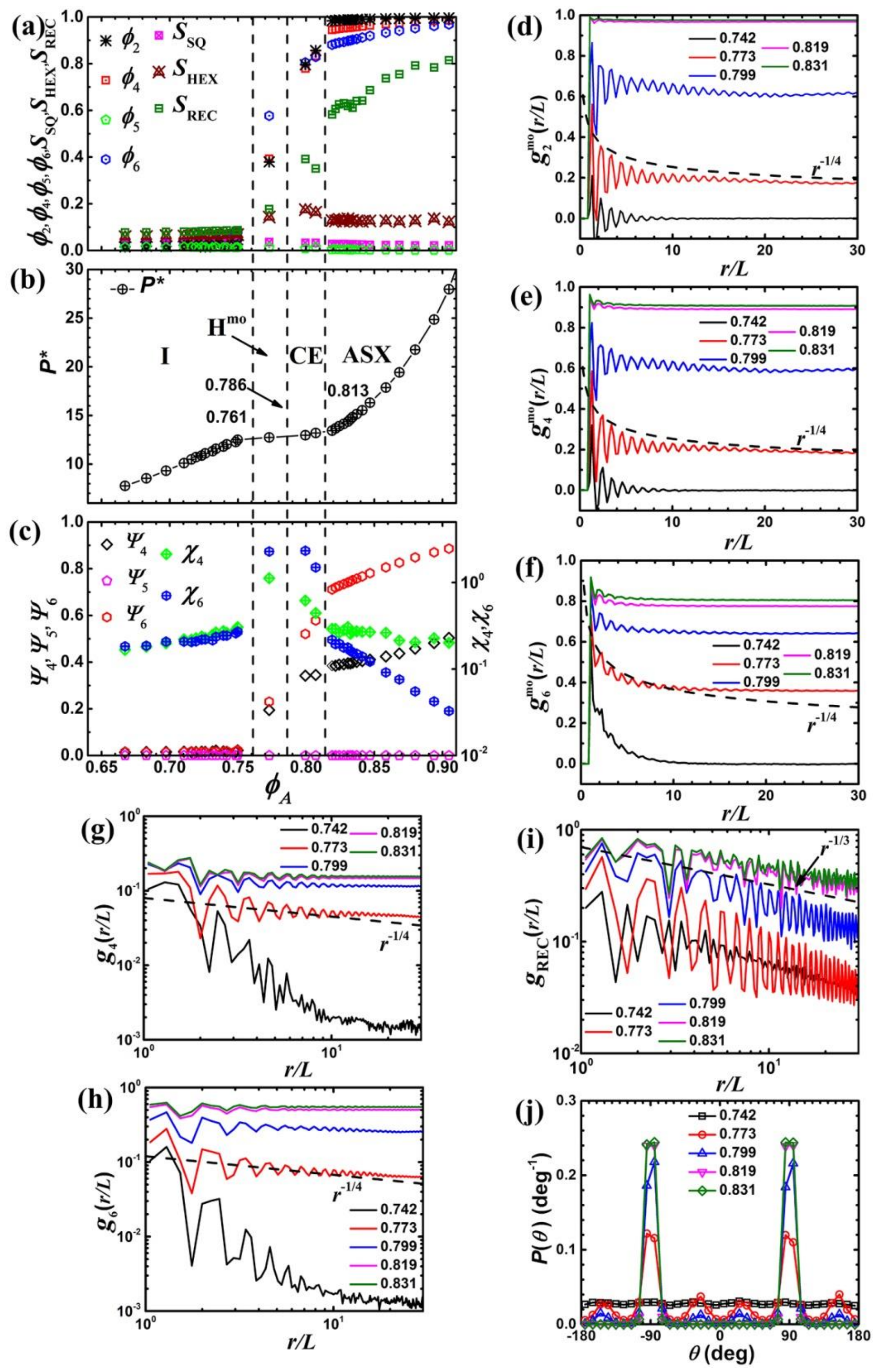

Supplementary Figure 4. Order parameters and correlation functions for different phases of kites of α = 60° obtained by *NPT* simulations. (a) Molecular-orientational order parameters, ϕ_2, ϕ_4, ϕ_5, and ϕ_6, positional order parameters of square lattice S_{SQ}, hexagonal lattice S_{HEX}, and rectangular lattice (complex lattice) S_{REC}; (b) Equation of state (EOS) with reduced pressure $P^* = PL^2/k_BT$; (c) Bond-orientational order parameters, Ψ_4, Ψ_5 and Ψ_6, and

susceptibilities of bond-orientational order parameters χ_4 and χ_6. Vertical dashed-lines are used to delimit different phases; (d-f) Molecular-orientational correlation functions, $g_2^{mo}(r)$, $g_4^{mo}(r)$ and $g_6^{mo}(r)$; (g-h) Bond-orientational correlation functions, $g_4(r)$ and $g_6(r)$; (i) Spatial correlation function of rectangular lattice, $g_{REC}(r)$; (j) Distribution $P(\theta)$ of single-particle orientation. Dashed lines in (d-i) are curves $\propto r^{-1/4}$ or $r^{-1/3}$.

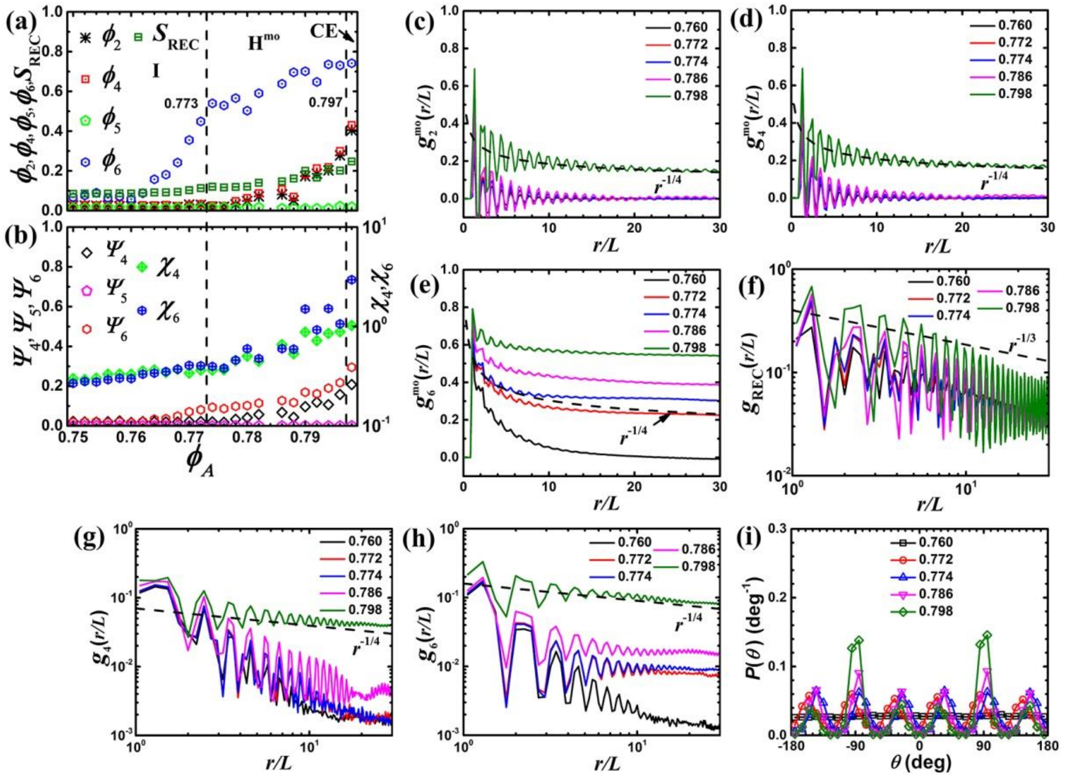

Supplementary Figure 5. Order parameters and correlation functions for different phases of kites of α = 60° obtained by *NVT* simulations. (a) Molecular-orientational order parameters, ϕ_2, ϕ_4, ϕ_5, and ϕ_6, positional order parameters of rectangular lattice (complex lattice) S_{REC}; (b) Bond-orientational order parameters, Ψ_4, Ψ_5 and Ψ_6, and susceptibilities of bond-orientational order parameters χ_4 and χ_6. Vertical dashed-lines are used to delimit different phases; (c-e) Molecular-orientational correlation functions, $g_2^{mo}(r)$, $g_4^{mo}(r)$ and $g_6^{mo}(r)$; (f) Spatial correlation functions of rectangular lattice, $g_{REC}(r)$; (g-h) Bond-orientational correlation functions, $g_4(r)$ and $g_6(r)$; (i) Distribution $P(\theta)$ of single-particle orientation. Dashed lines in (c-h) are curves $\propto r^{-1/4}$ or $r^{-1/3}$.

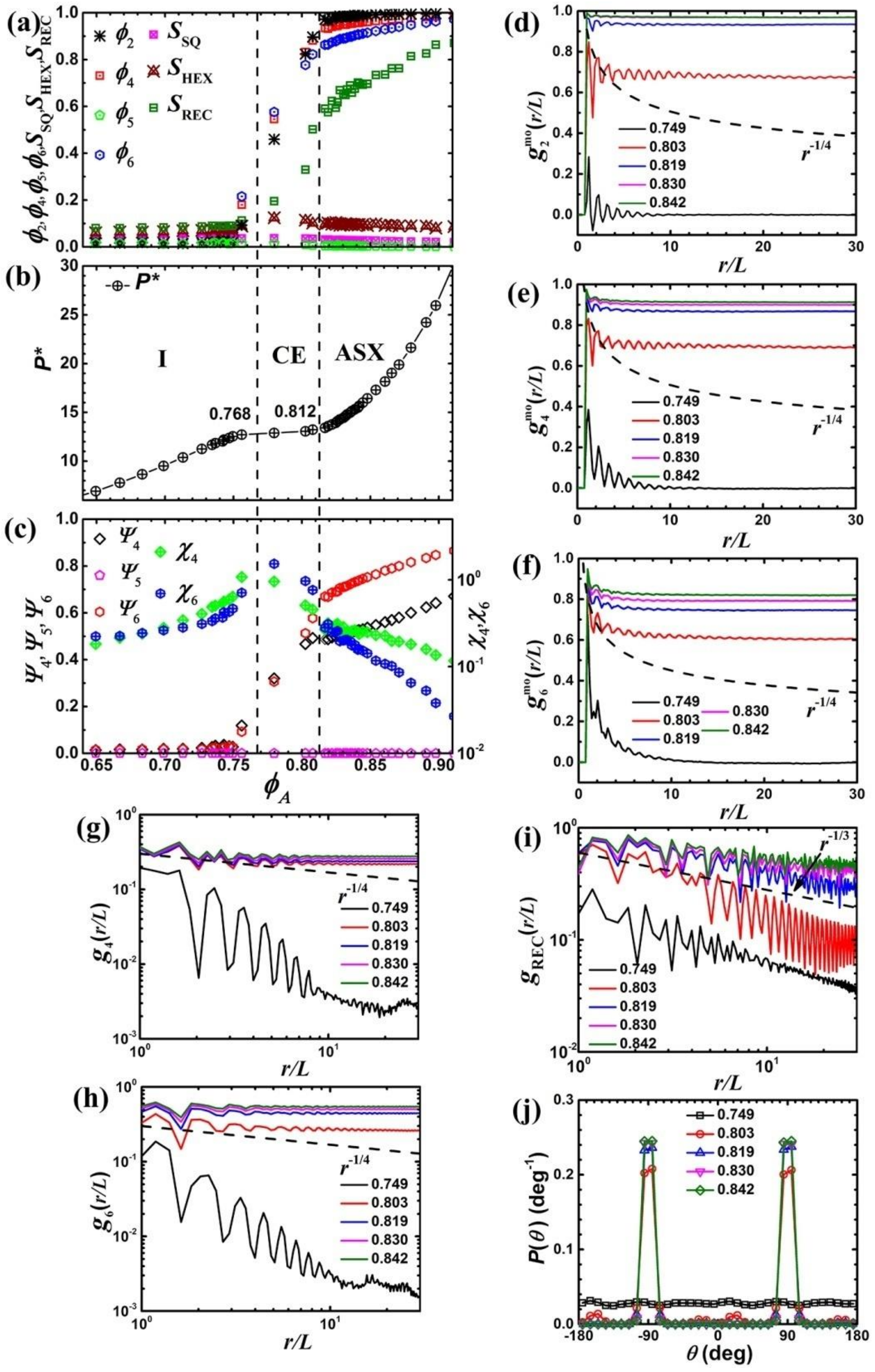

Supplementary Figure 6. Order parameters and correlation functions for different phases of kites of α = 66° obtained by *NPT* simulations. (a) Molecular-orientational order parameters, ϕ_2, ϕ_4, ϕ_5, and ϕ_6, positional order parameters of square lattice S_{SQ}, hexagonal lattice S_{HEX}, and rectangular lattice (complex lattice) S_{REC}; (b) Equation of state (EOS) with reduced pressure $P^* = PL^2/k_BT$; (c) Bond-orientational order parameters, Ψ_4, Ψ_5 and Ψ_6, and

susceptibilities of bond-orientational order parameters χ_4 and χ_6. Vertical dashed-lines are used to delimit different phases; (d-f) Molecular-orientational correlation functions, $g_2^{mo}(r)$, $g_4^{mo}(r)$ and $g_6^{mo}(r)$; (g-h) Bond-orientational correlation functions, $g_4(r)$ and $g_6(r)$; (i) Spatial correlation function of rectangular lattice, $g_{REC}(r)$; (j) Distribution $P(\theta)$ of single-particle orientation. Dashed lines in (d-i) are curves $\propto r^{-1/4}$ or $r^{-1/3}$.

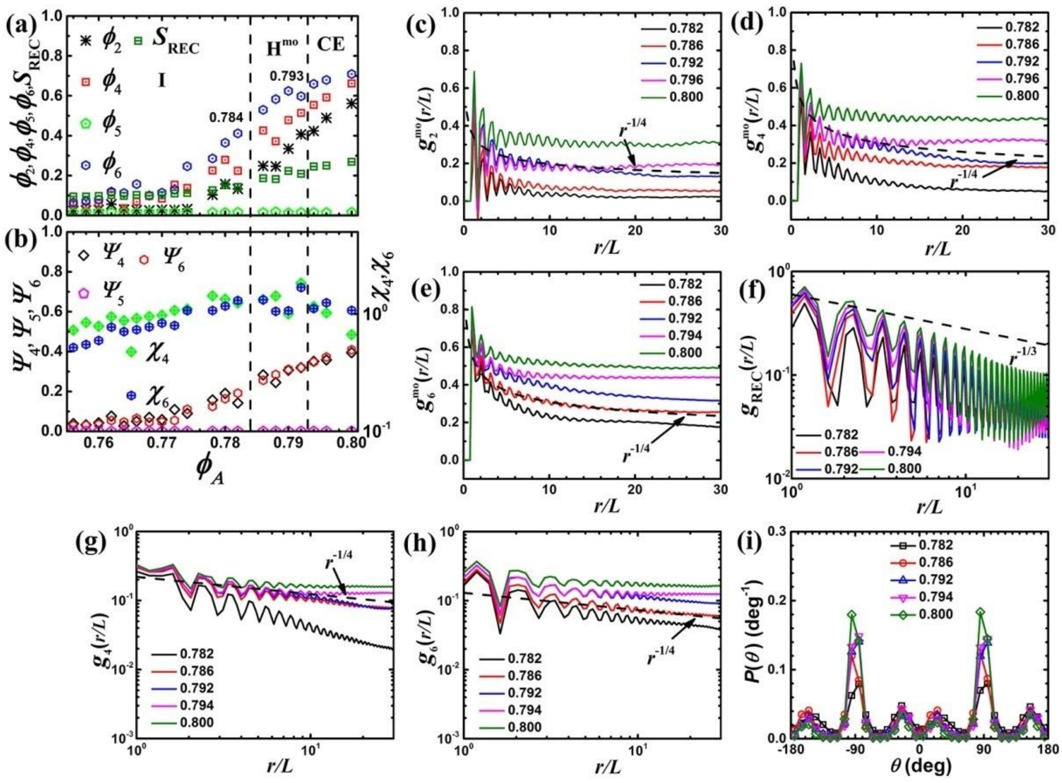

Supplementary Figure 7. Order parameters and correlation functions for different phases of kites of α = 66° obtained by *NVT* simulations. (a) Molecular-orientational order parameters, ϕ_2, ϕ_4, ϕ_5, and ϕ_6, positional order parameters of rectangular lattice (complex lattice) S_{REC}; (b) Bond-orientational order parameters, Ψ_4, Ψ_5 and Ψ_6, and susceptibilities of bond orientational order parameters χ_4 and χ_6. Vertical dashed-lines are used to delimit different phases; (c-e) Molecular-orientational correlation functions, $g_2^{mo}(r)$, $g_4^{mo}(r)$ and $g_6^{mo}(r)$; (f) Spatial correlation functions of rectangular lattice, $g_{REC}(r)$; (g-h) Bond-orientational correlation functions, $g_4(r)$ and $g_6(r)$; (i) Distribution $P(\theta)$ of single-particle orientation. Dashed lines in (c-h) are curves $\propto r^{-1/4}$ or $r^{-1/3}$.

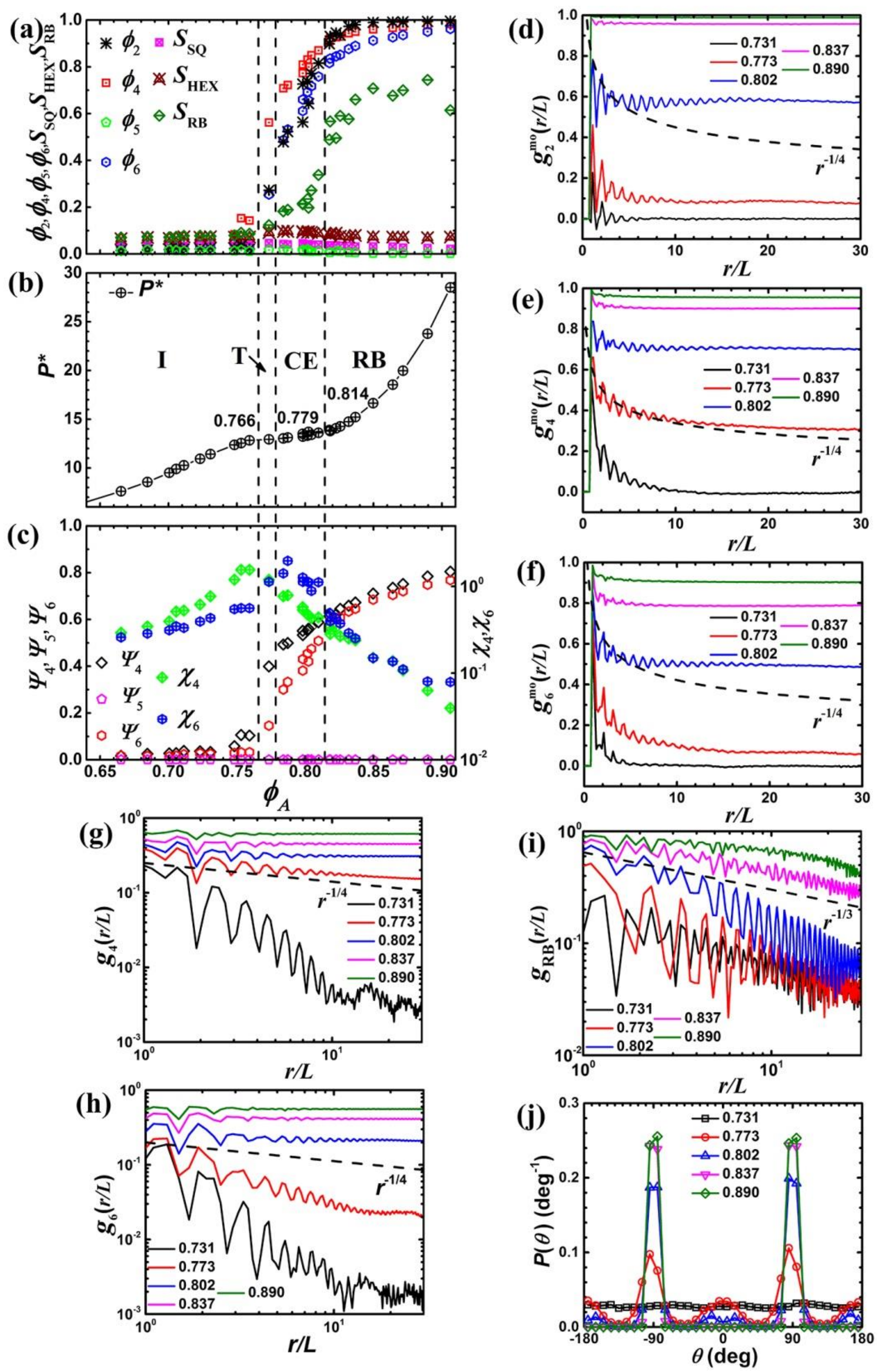

Supplementary Figure 8. Order parameters and correlation functions for different phases of kites of α = 72° obtained by *NPT* simulations. (a) Molecular-orientational order parameters, ϕ_2, ϕ_4, ϕ_5, and ϕ_6, positional order parameters of square lattice S_{SQ}, hexagonal lattice S_{HEX}, and rhombic lattice S_{RB}; (b) Equation of state (EOS) with reduced pressure $P^* = PL^2/k_BT$; (c) Bond-orientational order parameters, Ψ_4, Ψ_5 and Ψ_6, and susceptibilities of bond

orientational order parameters χ_4 and χ_6. Vertical dashed-lines are used to delimit different phases; (d-f) Molecular-orientational correlation functions, $g_2^{mo}(r)$, $g_4^{mo}(r)$ and $g_6^{mo}(r)$; (g-h) Bond-orientational correlation functions, $g_4(r)$ and $g_6(r)$; (i) Spatial correlation function of rhombic lattice, $g_{RB}(r)$; (j) Distribution $P(\theta)$ of single-particle orientation. Dashed lines in (d-i) are curves $\propto r^{-1/4}$ or $r^{-1/3}$.

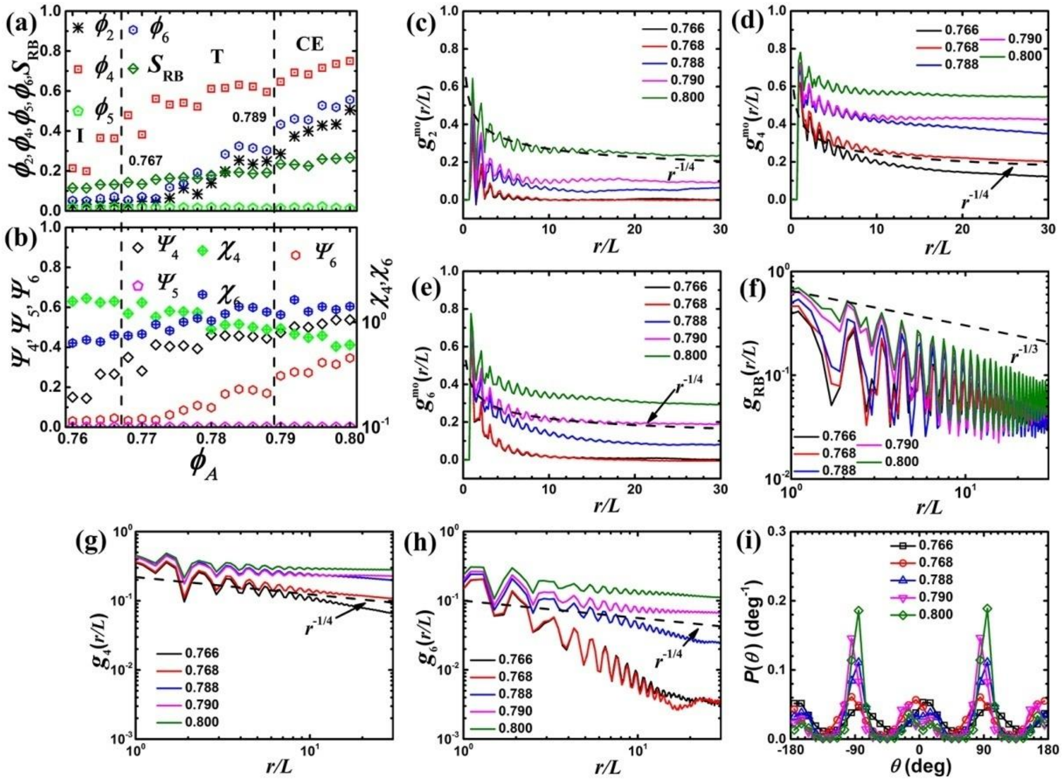

Supplementary Figure 9. Order parameters and correlation functions for different phases of kites of α = 72° obtained by *NVT* simulations. (a) Molecular-orientational order parameters, ϕ_2, ϕ_4, ϕ_5, and ϕ_6, positional order parameters of rhombic lattice S_{RB}; (b) Bond-orientational order parameters, Ψ_4, Ψ_5 and Ψ_6, and susceptibilities of bond orientational order parameters χ_4 and χ_6. Vertical dashed-lines are used to delimit different phases; (c-e) Molecular-orientational correlation functions, $g_2^{mo}(r)$, $g_4^{mo}(r)$ and $g_6^{mo}(r)$; (f) Spatial correlation functions of rhombic lattice, $g_{RB}(r)$; (g-h) Bond-orientational correlation functions, $g_4(r)$ and $g_6(r)$; (i) Distribution $P(\theta)$ of single-particle orientation. Dashed lines in (c-h) are curves $\propto r^{-1/4}$ or $r^{-1/3}$.

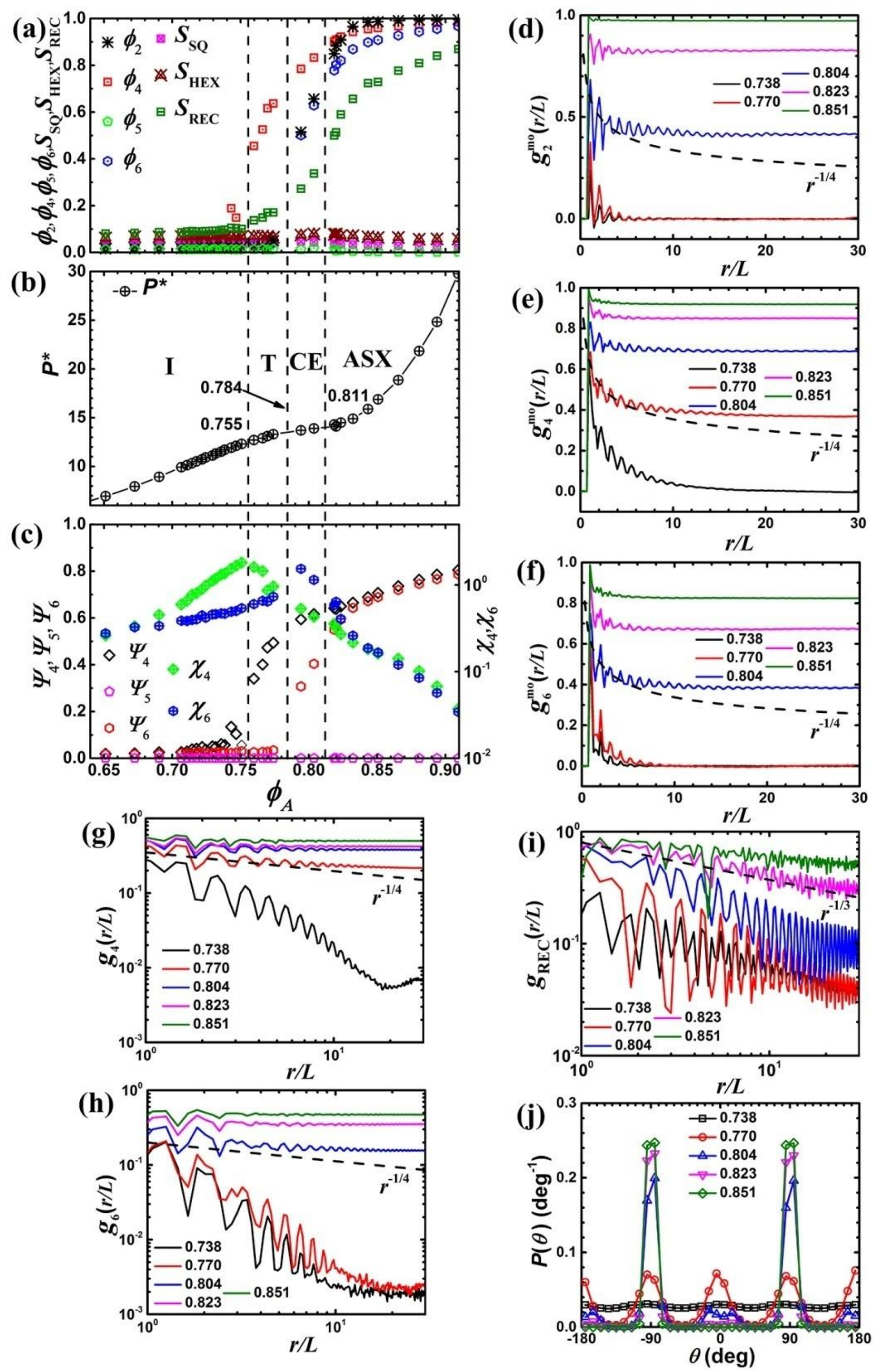

Supplementary Figure 10. Order parameters and correlation functions for different phases of kites of α = 75° obtained by *NPT* simulations. (a) Molecular-orientational order parameters, ϕ_2, ϕ_4, ϕ_5, and ϕ_6, positional order parameters of square lattice S_{SQ}, hexagonal lattice S_{HEX}, and rectangular lattice (complex lattice) S_{REC}; (b) Equation of state (EOS) with reduced pressure $P^* = PL^2/k_BT$; (c) Bond-orientational order parameters, Ψ_4, Ψ_5 and Ψ_6, and

susceptibilities of bond-orientational order parameters χ_4 and χ_6. Vertical dashed-lines are used to delimit different phases; (d-f) Molecular-orientational correlation functions, $g_2^{mo}(r)$, $g_4^{mo}(r)$ and $g_6^{mo}(r)$; (g-h) Bond-orientational correlation functions, $g_4(r)$ and $g_6(r)$; (i) Spatial correlation function of rectangular lattice, $g_{REC}(r)$; (j) Distribution $P(\theta)$ of single-particle orientation. Dashed lines in (d-i) are curves $\propto r^{-1/4}$ or $r^{-1/3}$.

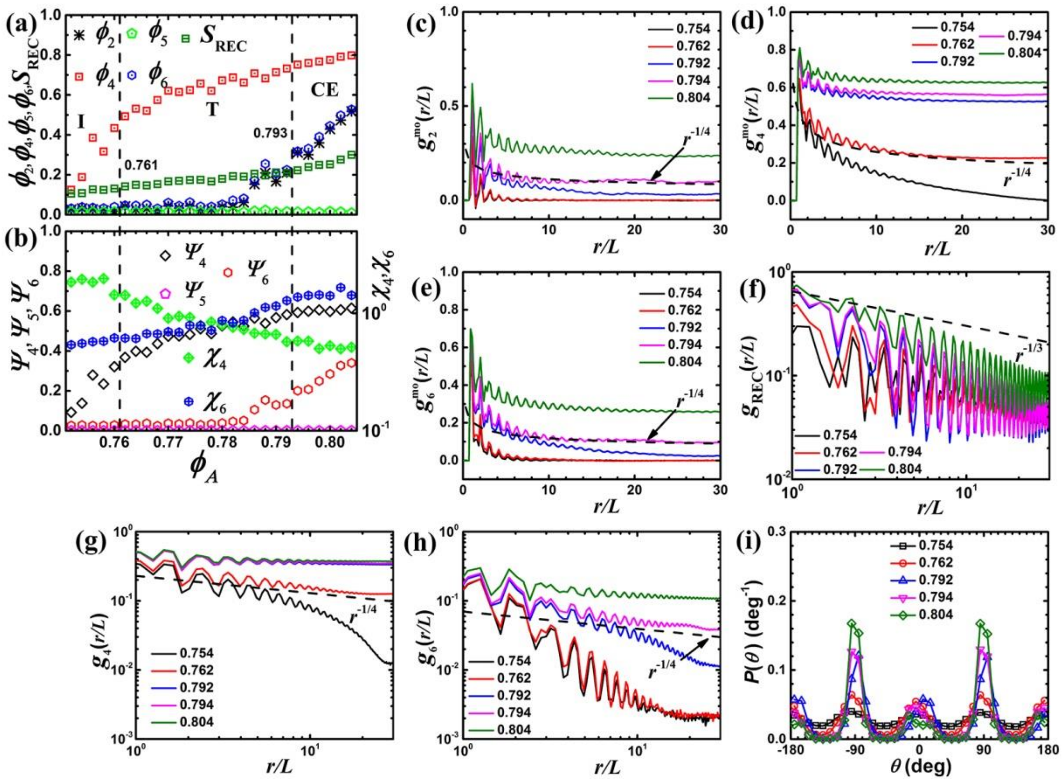

Supplementary Figure 11. Order parameters and correlation functions for different phases of kites of α = 75° obtained by *NVT* simulations. (a) Molecular-orientational order parameters, ϕ_2, ϕ_4, ϕ_5, and ϕ_6, positional order parameters of rectangular lattice (complex lattice) S_{REC}; (b) Bond-orientational order parameters, Ψ_4, Ψ_5 and Ψ_6, and susceptibilities of bond-orientational order parameters χ_4 and χ_6. Vertical dashed-lines are used to delimit different phases; (c-e) Molecular-orientational correlation functions, $g_2^{mo}(r)$, $g_4^{mo}(r)$ and $g_6^{mo}(r)$; (f) Spatial correlation functions of rectangular lattice, $g_{REC}(r)$; (g-h) Bond-orientational correlation functions, $g_4(r)$ and $g_6(r)$; (i) Distribution $P(\theta)$ of single-particle orientation. Dashed lines in (c-h) are curves $\propto r^{-1/4}$ or $r^{-1/3}$.

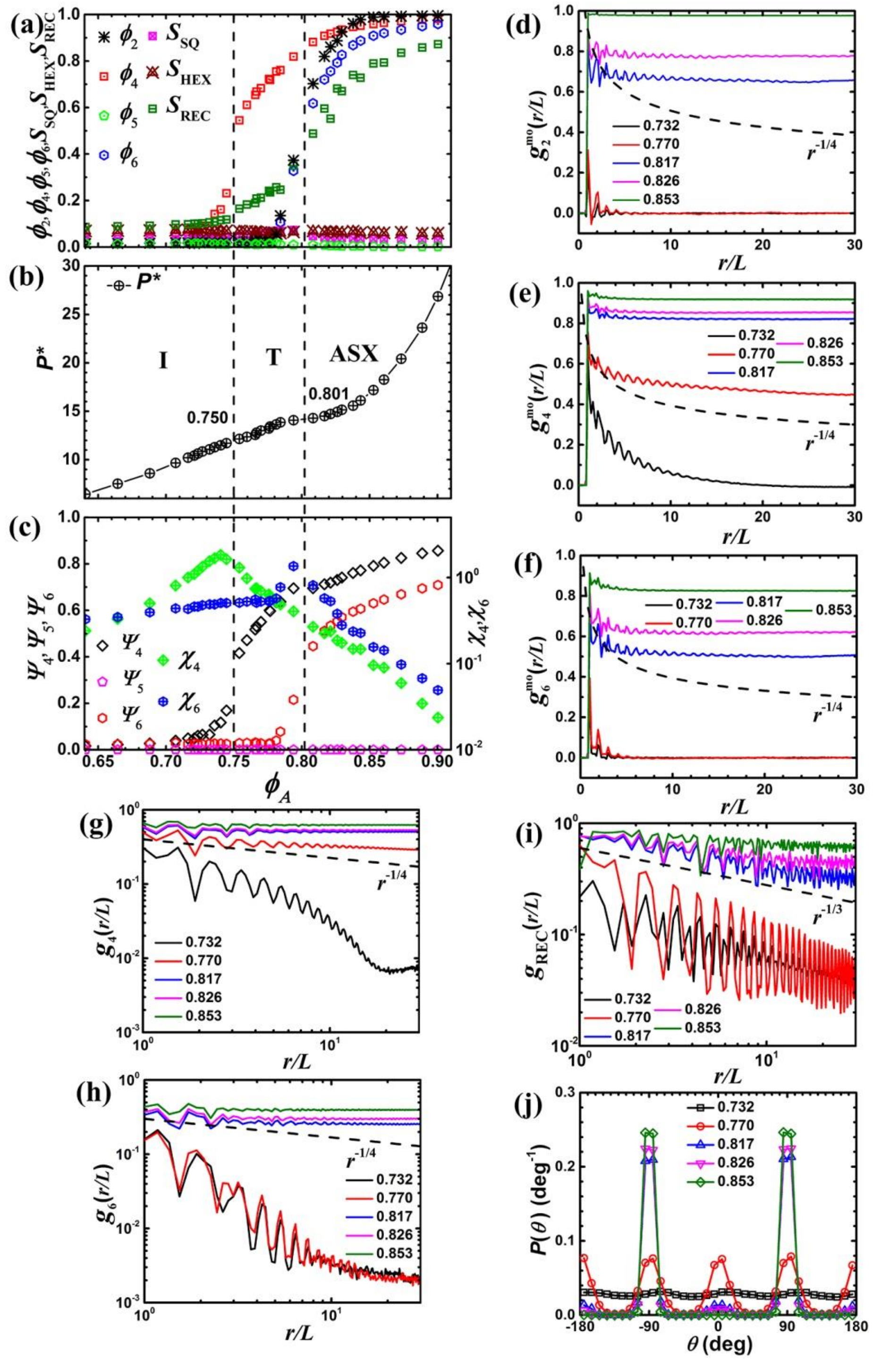

Supplementary Figure 12. Order parameters and correlation functions for different phases of kites of α = 81° obtained by *NPT* simulations. (a) Molecular-orientational order parameters, ϕ_2, ϕ_4, ϕ_5, and ϕ_6, positional order parameters of square lattice S_{SQ}, hexagonal lattice S_{HEX}, and rectangular lattice (complex lattice) S_{REC}; (b) Equation of state (EOS) with reduced pressure $P^* = PL^2/k_BT$; (c) Bond-orientational order parameters, Ψ_4, Ψ_5 and Ψ_6, and

susceptibilities of bond-orientational order parameters χ_4 and χ_6. Vertical dashed-lines are used to delimit different phases; (d-f) Molecular-orientational correlation functions, $g_2^{mo}(r)$, $g_4^{mo}(r)$ and $g_6^{mo}(r)$; (g-h) Bond-orientational correlation functions, $g_4(r)$ and $g_6(r)$; (i) Spatial correlation function of rectangular lattice, $g_{REC}(r)$; (j) Distribution $P(\theta)$ of single-particle orientation. Dashed lines in (d-i) are curves $\propto r^{-1/4}$ or $r^{-1/3}$.

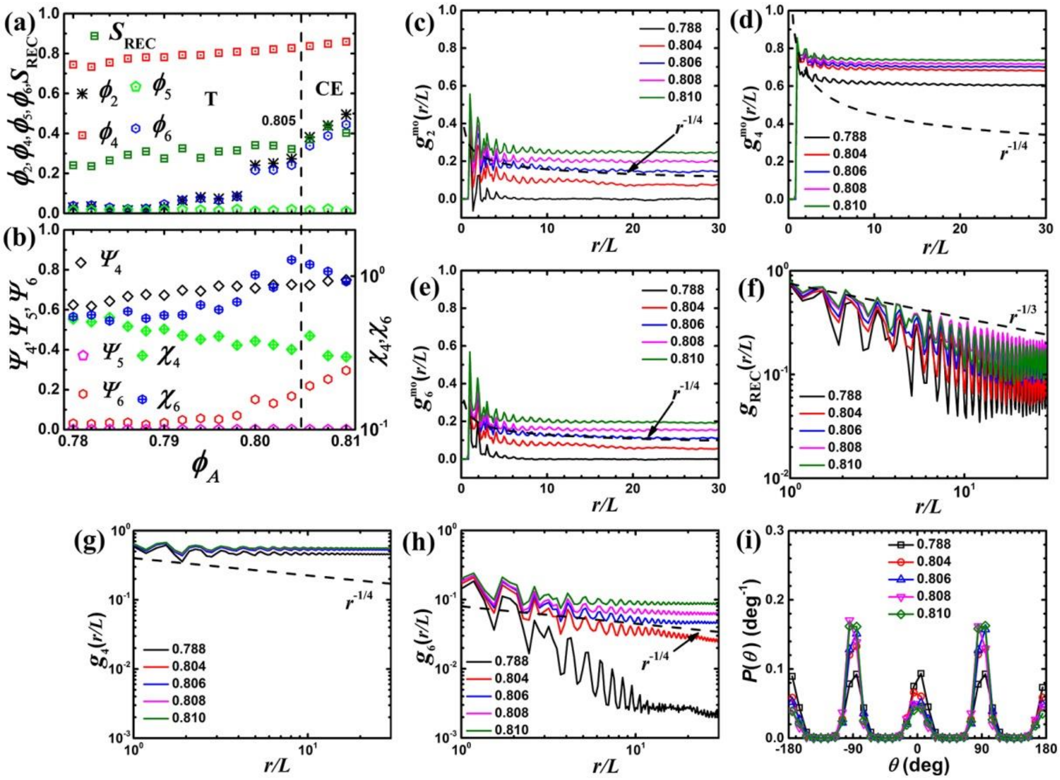

Supplementary Figure 13. Order parameters and correlation functions for different phases of kites of α = 81° obtained by *NVT* simulations. (a) Molecular-orientational order parameters, ϕ_2, ϕ_4, ϕ_5, and ϕ_6, positional order parameters of rectangular lattice (complex lattice) S_{REC}; (b) Bond-orientational order parameters, Ψ_4, Ψ_5 and Ψ_6, and susceptibilities of bond-orientational order parameters χ_4 and χ_6. Vertical dashed-lines are used to delimit different phases; (c-e) Molecular-orientational correlation functions, $g_2^{mo}(r)$, $g_4^{mo}(r)$ and $g_6^{mo}(r)$; (f) Spatial correlation functions of rectangular lattice, $g_{REC}(r)$; (g-h) Bond-orientational correlation functions, $g_4(r)$ and $g_6(r)$; (i) Distribution $P(\theta)$ of single-particle orientation. Dashed lines in (c-h) are curves $\propto r^{-1/4}$ or $r^{-1/3}$.

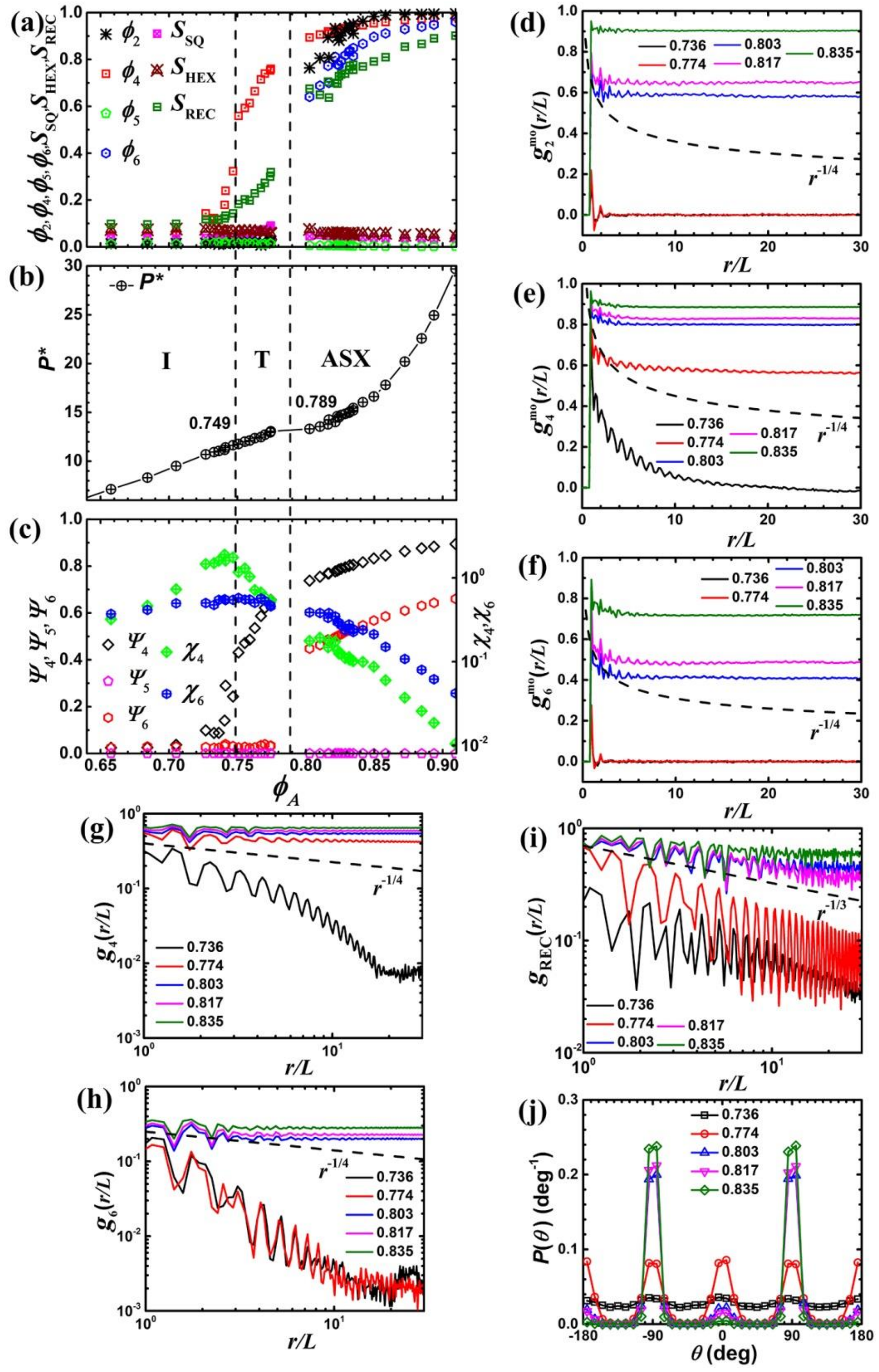

Supplementary Figure 14. Order parameters and correlation functions for different phases of kites of α = 90° obtained by *NPT* simulations. (a) Molecular-orientational order parameters, ϕ_2, ϕ_4, ϕ_5, and ϕ_6, positional order parameters of square lattice S_{SQ}, hexagonal lattice S_{HEX}, and rectangular lattice (complex lattice) S_{REC}; (b) Equation of state (EOS) with reduced pressure $P^* = PL^2/k_BT$; (c) Bond-orientational order parameters, Ψ_4, Ψ_5 and Ψ_6, and

susceptibilities of bond-orientational order parameters χ_4 and χ_6. Vertical dashed-lines are used to delimit different phases; (d-f) Molecular-orientational correlation functions, $g_2^{mo}(r)$, $g_4^{mo}(r)$ and $g_6^{mo}(r)$; (g-h) Bond-orientational correlation functions, $g_4(r)$ and $g_6(r)$; (i) Spatial correlation function of rectangular lattice, $g_{REC}(r)$; (j) Distribution $P(\theta)$ of single-particle orientation. Dashed lines in (d-i) are curves $\propto r^{-1/4}$ or $r^{-1/3}$.

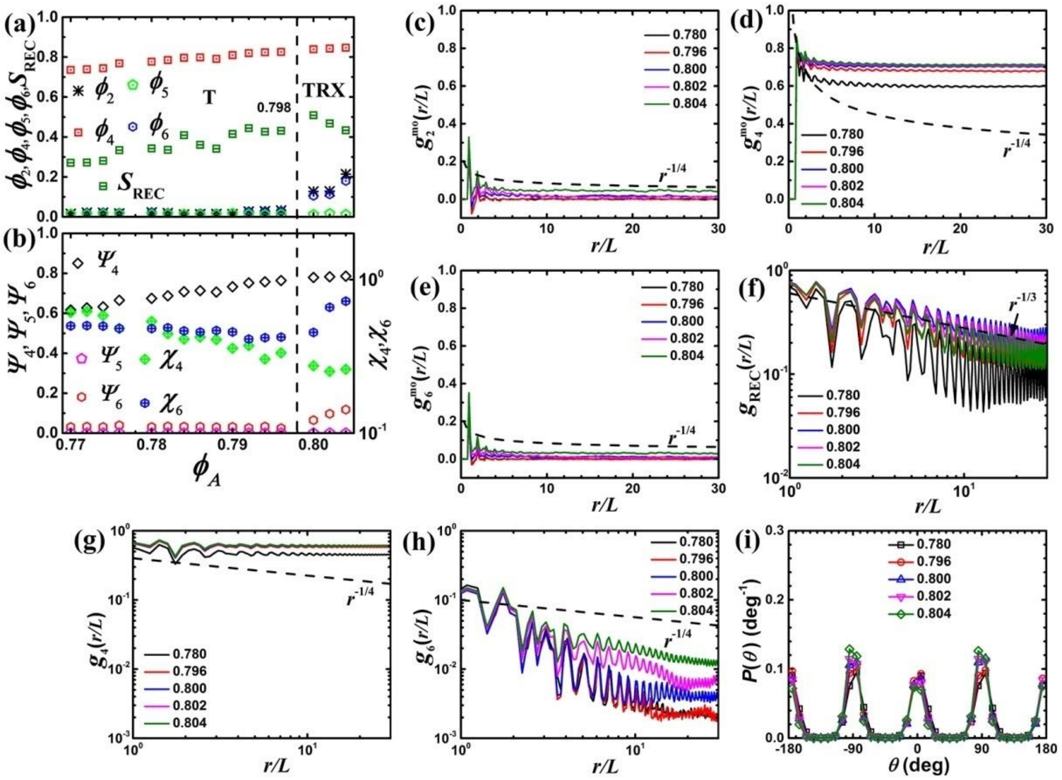

Supplementary Figure 15. Order parameters and correlation functions for different phases of kites of α = 90° obtained by *NVT* simulations. (a) Molecular-orientational order parameters, ϕ_2, ϕ_4, ϕ_5, and ϕ_6, positional order parameters of rectangular lattice (complex lattice) S_{REC}; (b) Bond-orientational order parameters, Ψ_4, Ψ_5 and Ψ_6, and susceptibilities of bond-orientational order parameters χ_4 and χ_6. Vertical dashed-lines are used to delimit different phases; (c-e) Molecular-orientational correlation functions, $g_2^{mo}(r)$, $g_4^{mo}(r)$ and $g_6^{mo}(r)$; (f) Spatial correlation functions of rectangular lattice, $g_{REC}(r)$; (g-h) Bond-orientational correlation functions, $g_4(r)$ and $g_6(r)$; (i) Distribution $P(\theta)$ of single-particle orientation. Dashed lines in (c-h) are curves $\propto r^{-1/4}$ or $r^{-1/3}$.

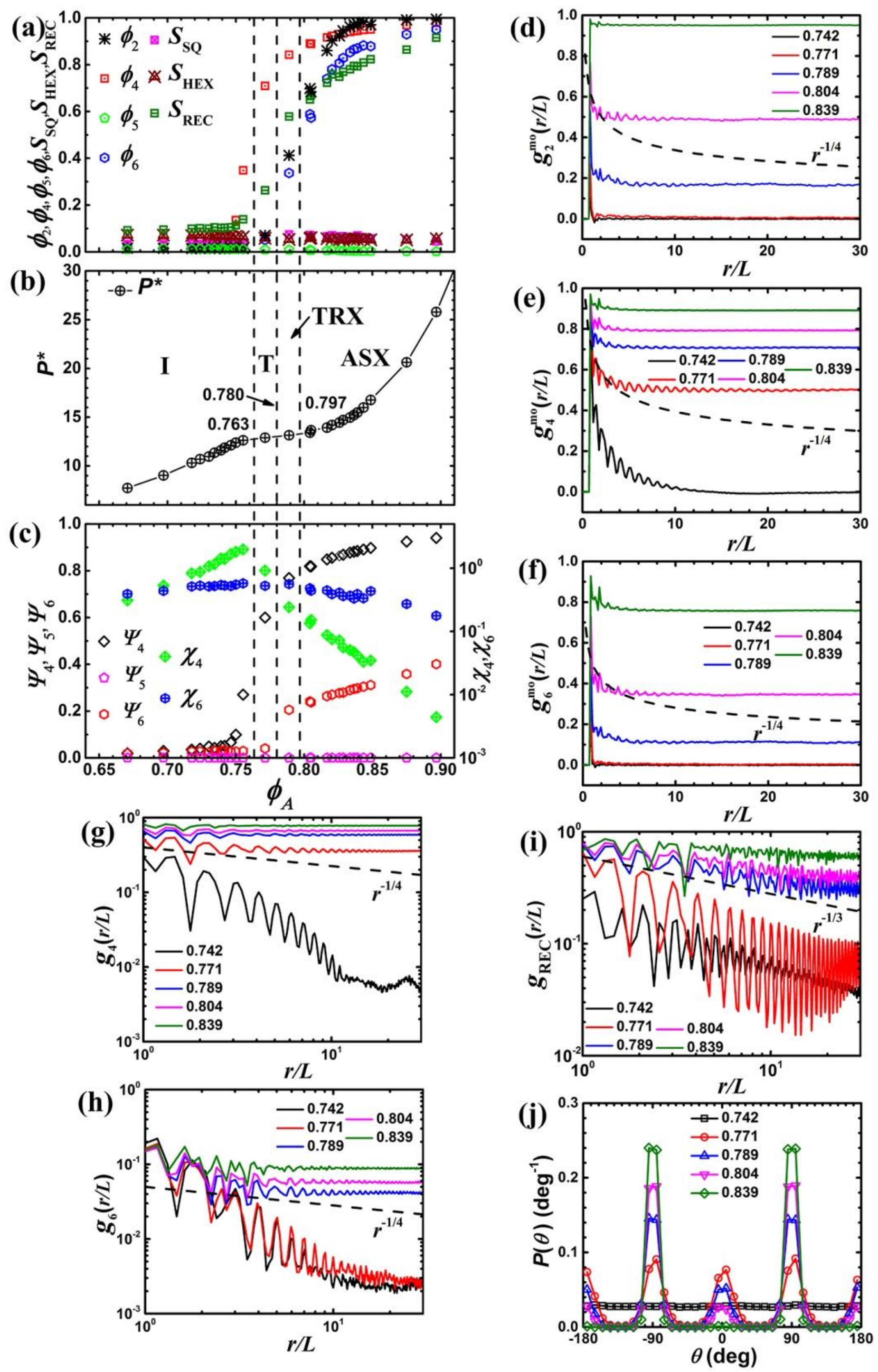

Supplementary Figure 16. Order parameters and correlation functions for different phases of kites of α = 99° obtained by *NPT* simulations using a square box. (a) Molecular-orientational order parameters, ϕ_2, ϕ_4, ϕ_5, and ϕ_6, positional order parameters of square lattice S_{SQ}, hexagonal lattice S_{HEX}, and rectangular lattice (complex lattice) S_{REC}; (b) Equation of state (EOS) with reduced pressure $P^* = PL^2/k_BT$; (c) Bond-orientational order parameters, Ψ_4, Ψ_5 and Ψ_6, and

susceptibilities of bond-orientational order parameters χ_4 and χ_6. Vertical dashed-lines are used to delimit different phases; (d-f) Molecular-orientational correlation functions, $g_2^{mo}(r)$, $g_4^{mo}(r)$ and $g_6^{mo}(r)$; (g-h) Bond-orientational correlation functions, $g_4(r)$ and $g_6(r)$; (i) Spatial correlation function of rectangular lattice, $g_{REC}(r)$; (j) Distribution $P(\theta)$ of single-particle orientation. Dashed lines in (d-i) are curves $\propto r^{-1/4}$ or $r^{-1/3}$.

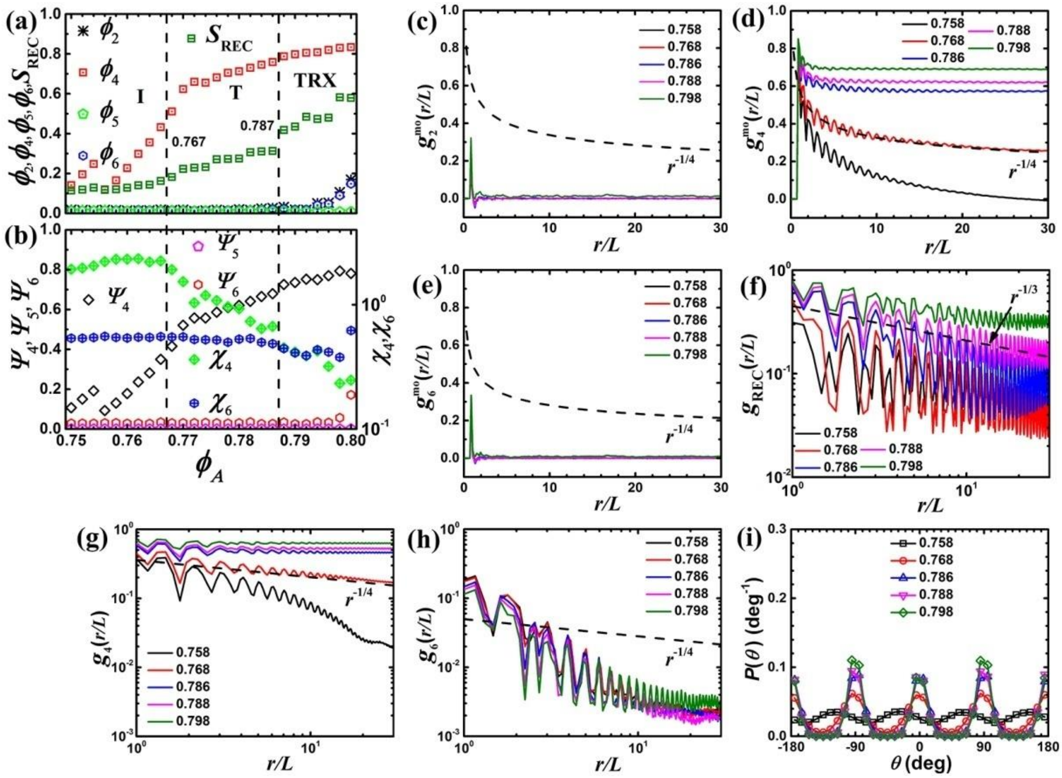

Supplementary Figure 17. Order parameters and correlation functions for different phases of kites of α = 99° obtained by *NVT* simulations. (a) Molecular-orientational order parameters, ϕ_2, ϕ_4, ϕ_5, and ϕ_6, positional order parameters of rectangular lattice (complex lattice) S_{REC}; (b) Bond-orientational order parameters, Ψ_4, Ψ_5 and Ψ_6, and susceptibilities of bond-orientational order parameters χ_4 and χ_6. Vertical dashed-lines are used to delimit different phases; (c-e) Molecular-orientational correlation functions, $g_2^{mo}(r)$, $g_4^{mo}(r)$ and $g_6^{mo}(r)$; (f) Spatial correlation functions of rectangular lattice, $g_{REC}(r)$; (g-h) Bond-orientational correlation functions, $g_4(r)$ and $g_6(r)$; (i) Distribution $P(\theta)$ of single-particle orientation. Dashed lines in (c-h) are curves $\propto r^{-1/4}$ or $r^{-1/3}$.

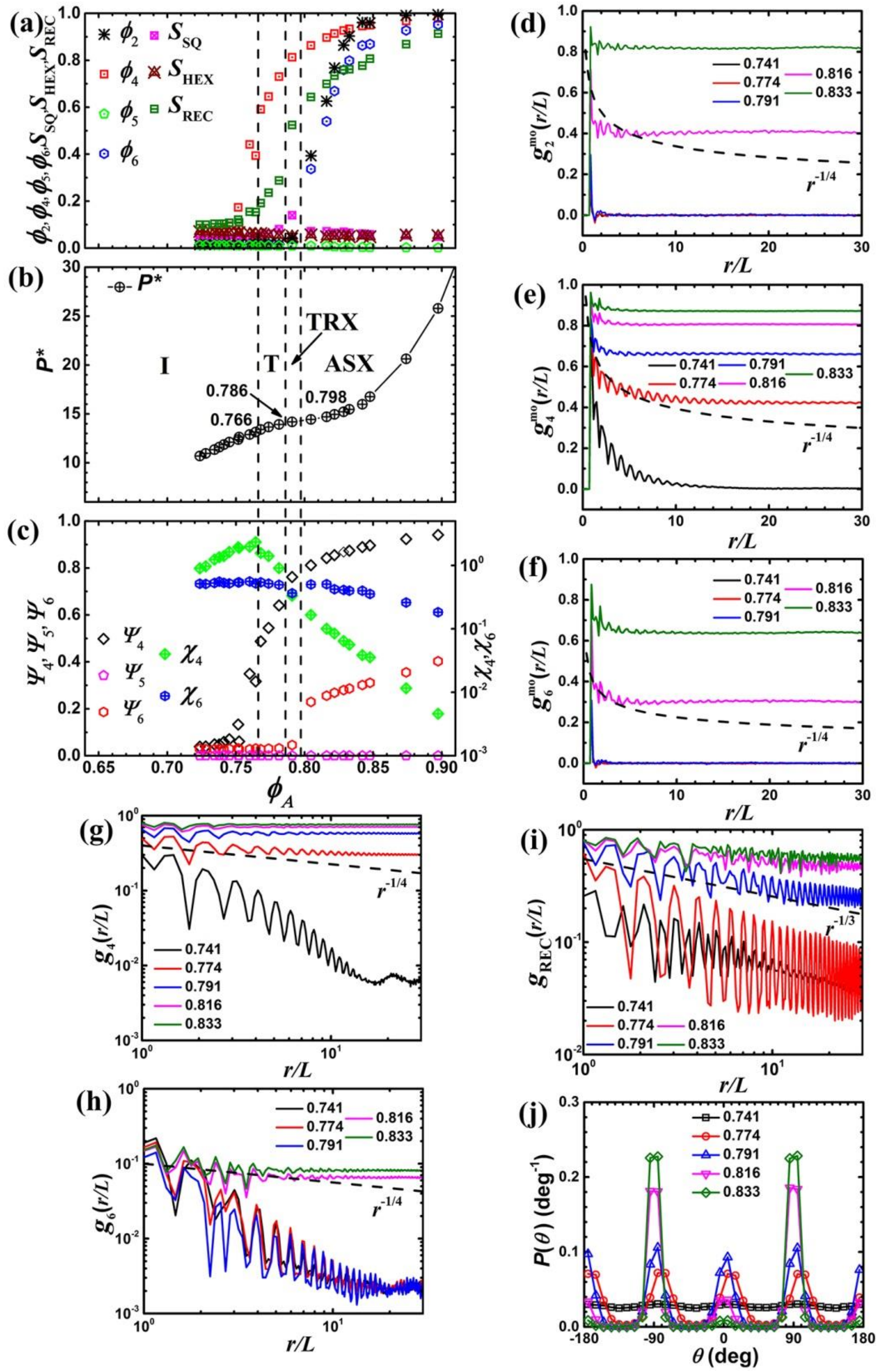

Supplementary Figure 18. Order parameters and correlation functions for different phases of kites of α = 99° obtained by $N\sigma_P T$ ensembles. (a) Molecular-orientational order parameters, ϕ_2, ϕ_4, ϕ_5, and ϕ_6, positional order parameters of square lattice S_{SQ}, hexagonal lattice S_{HEX}, and rectangular lattice (complex lattice) S_{REC}; (b) Equation of state (EOS) with reduced pressure $P^* = PL^2/k_B T$; (c) Bond-orientational order parameters, Ψ_4, Ψ_5 and Ψ_6, and

susceptibilities of bond-orientational order parameters χ_4 and χ_6. Vertical dashed-lines are used to delimit different phases; (d-f) Molecular-orientational correlation functions, $g_2^{mo}(r)$, $g_4^{mo}(r)$ and $g_6^{mo}(r)$; (g-h) Bond-orientational correlation functions, $g_4(r)$ and $g_6(r)$; (i) Spatial correlation function of rectangular lattice, $g_{REC}(r)$; (j) Distribution $P(\theta)$ of single-particle orientation. Dashed lines in (d-i) are curves $\propto r^{-1/4}$ or $r^{-1/3}$.

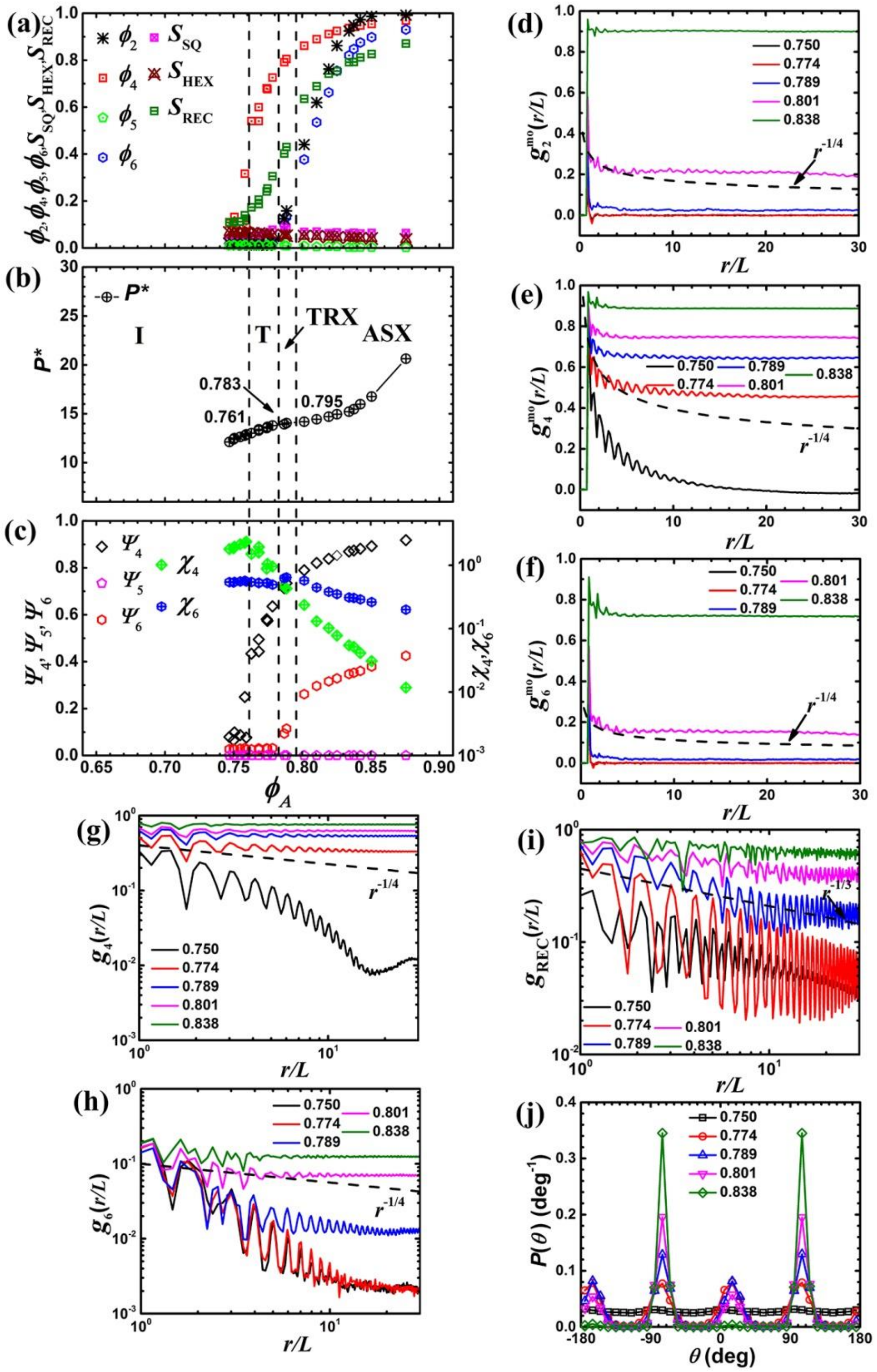

Supplementary Figure 19. Order parameters and correlation functions for different phases of kites of α = 99° obtained by *NPT* simulations using a 60° rhombic-shaped box. (a) Molecular-orientational order parameters, ϕ_2, ϕ_4, ϕ_5, and ϕ_6, positional order parameters of square lattice S_{SQ}, hexagonal lattice S_{HEX}, and rectangular lattice (complex lattice) S_{REC}; (b) Equation of state (EOS) with reduced pressure $P^* = PL^2/k_BT$; (c) Bond-orientational order parameters, Ψ_4, Ψ_5 and Ψ_6, and susceptibilities of bond-orientational order parameters χ_4 and χ_6. Vertical dashed-lines are used to delimit different phases; (d-f) Molecular-orientational correlation functions, $g_2^{mo}(r)$, $g_4^{mo}(r)$ and $g_6^{mo}(r)$; (g-h) Bond-orientational correlation functions, $g_4(r)$ and $g_6(r)$; (i) Spatial correlation function of rectangular lattice, $g_{REC}(r)$; (j) Distribution $P(\theta)$ of single-particle orientation. Dashed lines in (d-i) are curves $\propto$ $r^{-1/4}$ or $r^{-1/3}$.

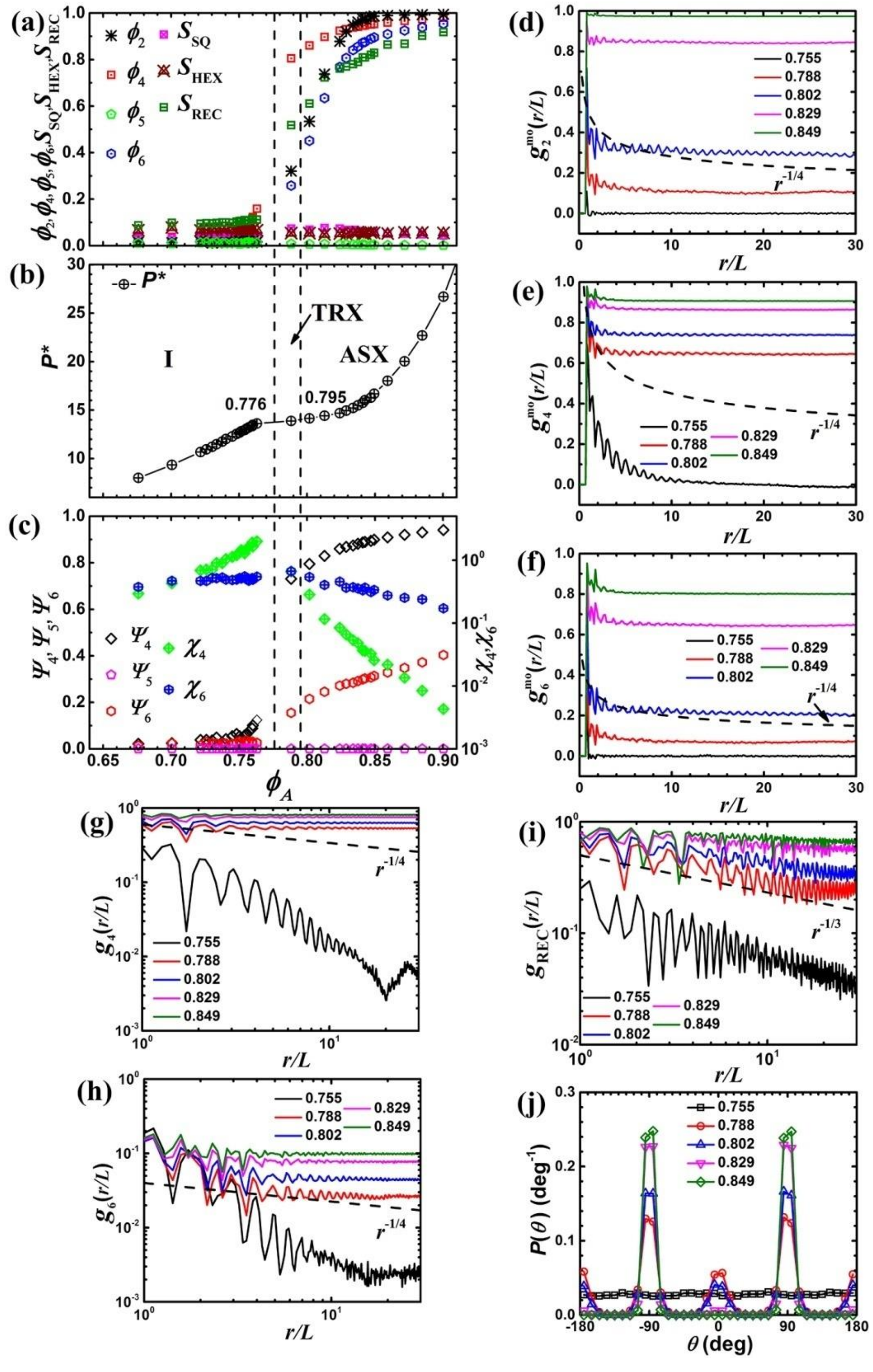

Supplementary Figure 20. Order parameters and correlation functions for different phases of kites of α = 103.5° obtained by *NPT* simulations. (a) Molecular-orientational order parameters, ϕ_2, ϕ_4, ϕ_5, and ϕ_6, positional order parameters of square lattice S_{SQ}, hexagonal lattice S_{HEX}, and rectangular lattice (complex lattice) S_{REC}; (b) Equation of state (EOS) with reduced pressure $P^* = PL^2/k_BT$; (c) Bond-orientational order parameters, Ψ_4, Ψ_5 and Ψ_6, and

susceptibilities of bond-orientational order parameters χ_4 and χ_6. Vertical dashed-lines are used to delimit different phases; (d-f) Molecular-orientational correlation functions, $g_2^{mo}(r)$, $g_4^{mo}(r)$ and $g_6^{mo}(r)$; (g-h) Bond-orientational correlation functions, $g_4(r)$ and $g_6(r)$; (i) Spatial correlation function of rectangular lattice, $g_{REC}(r)$; (j) Distribution $P(\theta)$ of single-particle orientation. Dashed lines in (d-i) are curves $\propto r^{-1/4}$ or $r^{-1/3}$.

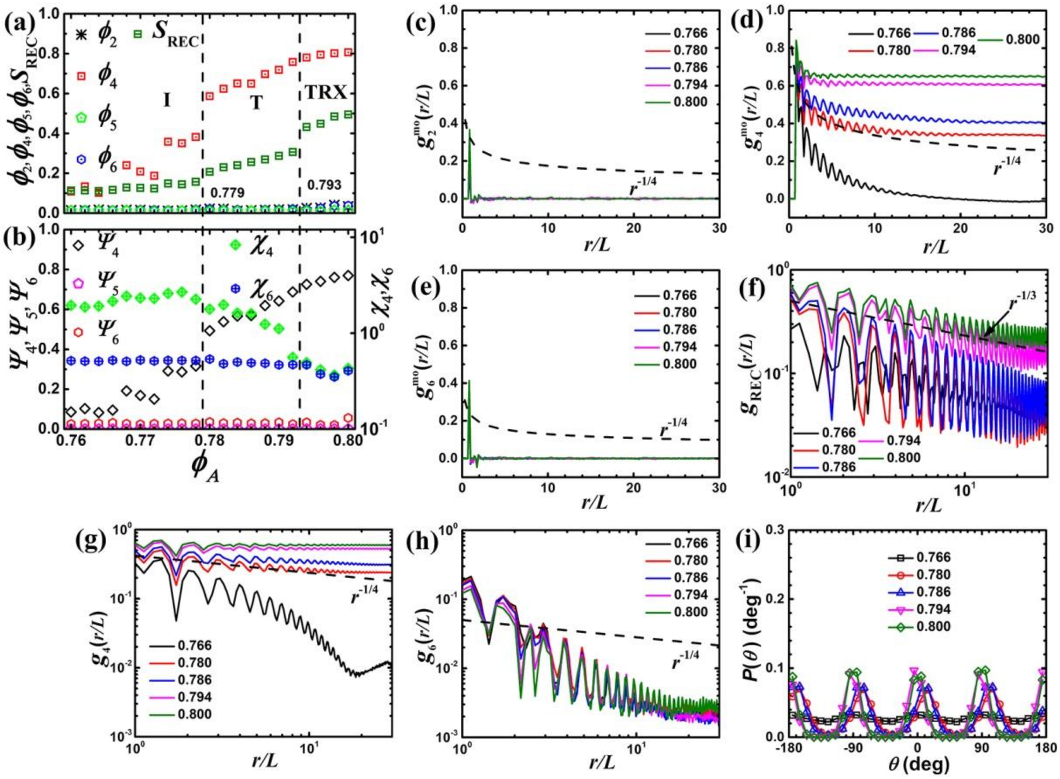

Supplementary Figure 21. Order parameters and correlation functions for different phases of kites of α = 103.5° obtained by *NVT* simulations. (a) Molecular-orientational order parameters, ϕ_2, ϕ_4, ϕ_5, and ϕ_6, positional order parameters of rectangular lattice (complex lattice) S_{REC}; (b) Bond-orientational order parameters, Ψ_4, Ψ_5 and Ψ_6, and susceptibilities of bond-orientational order parameters χ_4 and χ_6. Vertical dashed-lines are used to delimit different phases; (c-e) Molecular-orientational correlation functions, $g_2^{mo}(r)$, $g_4^{mo}(r)$ and $g_6^{mo}(r)$; (f) Spatial correlation functions of rectangular lattice, $g_{REC}(r)$; (g-h) Bond-orientational correlation functions, $g_4(r)$ and $g_6(r)$; (i) Distribution $P(\theta)$ of single-particle orientation. Dashed lines in (c-h) are curves $\propto r^{-1/4}$ or $r^{-1/3}$.

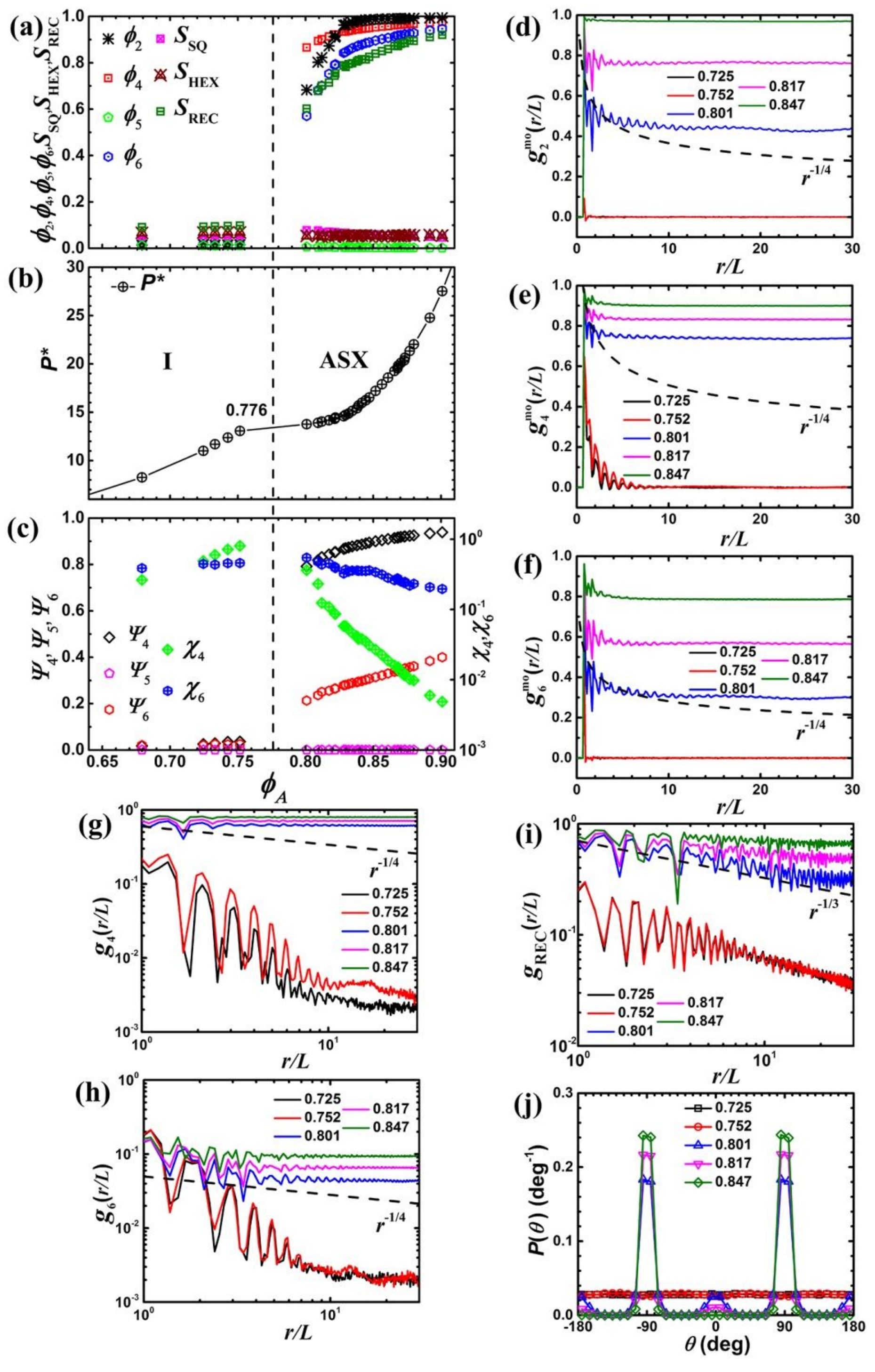

Supplementary Figure 22. Order parameters and correlation functions for different phases of kites of α = 108° obtained by *NPT* simulations. (a) Molecular-orientational order parameters, ϕ_2, ϕ_4, ϕ_5, and ϕ_6, positional order parameters of square lattice S_{SQ}, hexagonal lattice S_{HEX}, and rectangular lattice (complex lattice) S_{REC}; (b) Equation of state (EOS) with reduced pressure $P^* = PL^2/k_BT$; (c) Bond-orientational order parameters, Ψ_4, Ψ_5 and Ψ_6, and

susceptibilities of bond-orientational order parameters χ_4 and χ_6. Vertical dashed-lines are used to delimit different phases; (d-f) Molecular-orientational correlation functions, $g_2^{mo}(r)$, $g_4^{mo}(r)$ and $g_6^{mo}(r)$; (g-h) Bond-orientational correlation functions, $g_4(r)$ and $g_6(r)$; (i) Spatial correlation function of rectangular lattice, $g_{REC}(r)$; (j) Distribution $P(\theta)$ of single-particle orientation. Dashed lines in (d-i) are curves $\propto r^{-1/4}$ or $r^{-1/3}$.

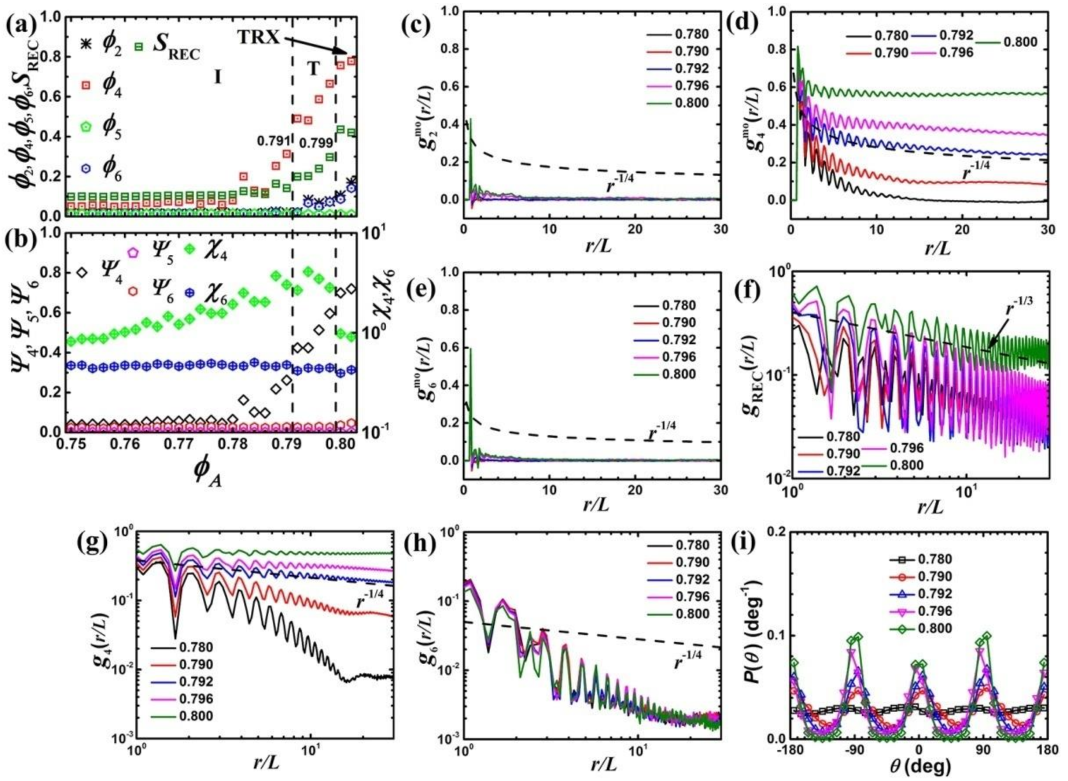

Supplementary Figure 23. Order parameters and correlation functions for different phases of kites of α = 108° obtained by *NVT* simulations. (a) Molecular-orientational order parameters, ϕ_2, ϕ_4, ϕ_5, and ϕ_6, positional order parameters of rectangular lattice (complex lattice) S_{REC}; (b) Bond-orientational order parameters, Ψ_4, Ψ_5 and Ψ_6, and susceptibilities of bond-orientational order parameters χ_4 and χ_6. Vertical dashed-lines are used to delimit different phases; (c-e) Molecular-orientational correlation functions, $g_2^{mo}(r)$, $g_4^{mo}(r)$ and $g_6^{mo}(r)$; (f) Spatial correlation functions of rectangular lattice, $g_{REC}(r)$; (g-h) Bond-orientational correlation functions, $g_4(r)$ and $g_6(r)$; (i) Distribution $P(\theta)$ of single-particle orientation. Dashed lines in (c-h) are curves $\propto r^{-1/4}$ or $r^{-1/3}$.

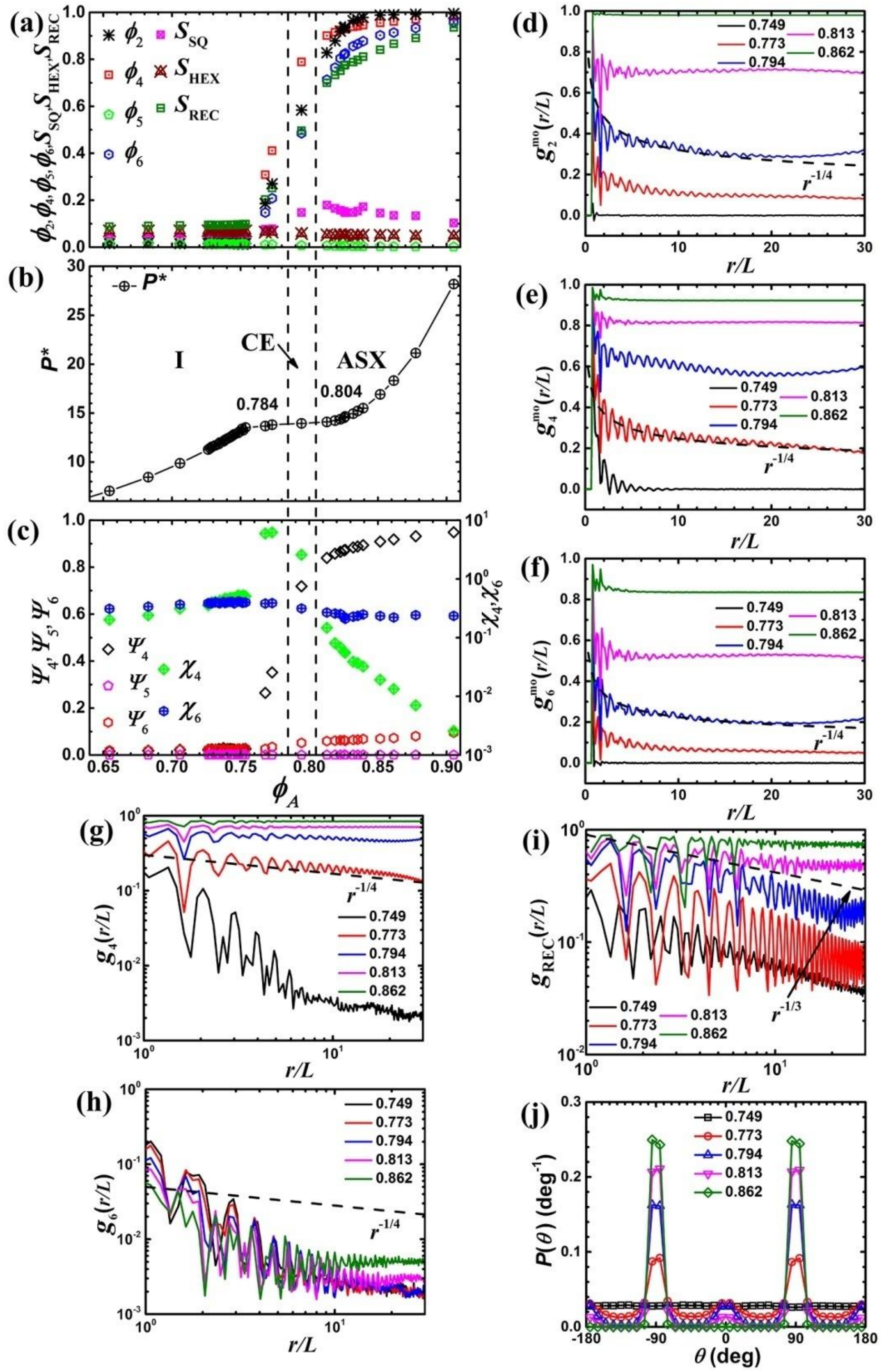

Supplementary Figure 24. Order parameters and correlation functions for different phases of kites of α = 112° obtained by *NPT* simulations. (a) Molecular-orientational order parameters, ϕ_2, ϕ_4, ϕ_5, and ϕ_6, positional order parameters of square lattice S_{SQ}, hexagonal lattice S_{HEX}, and rectangular lattice (complex lattice) S_{REC}; (b) Equation of state (EOS) with reduced pressure $P^* = PL^2/k_BT$; (c) Bond-orientational order parameters, Ψ_4, Ψ_5 and Ψ_6, and

susceptibilities of bond-orientational order parameters χ_4 and χ_6. Vertical dashed-lines are used to delimit different phases; (d-f) Molecular-orientational correlation functions, $g_2^{mo}(r)$, $g_4^{mo}(r)$ and $g_6^{mo}(r)$; (g-h) Bond-orientational correlation functions, $g_4(r)$ and $g_6(r)$; (i) Spatial correlation function of rectangular lattice, $g_{REC}(r)$; (j) Distribution $P(\theta)$ of single-particle orientation. Dashed lines in (d-i) are curves $\propto r^{-1/4}$ or $r^{-1/3}$.

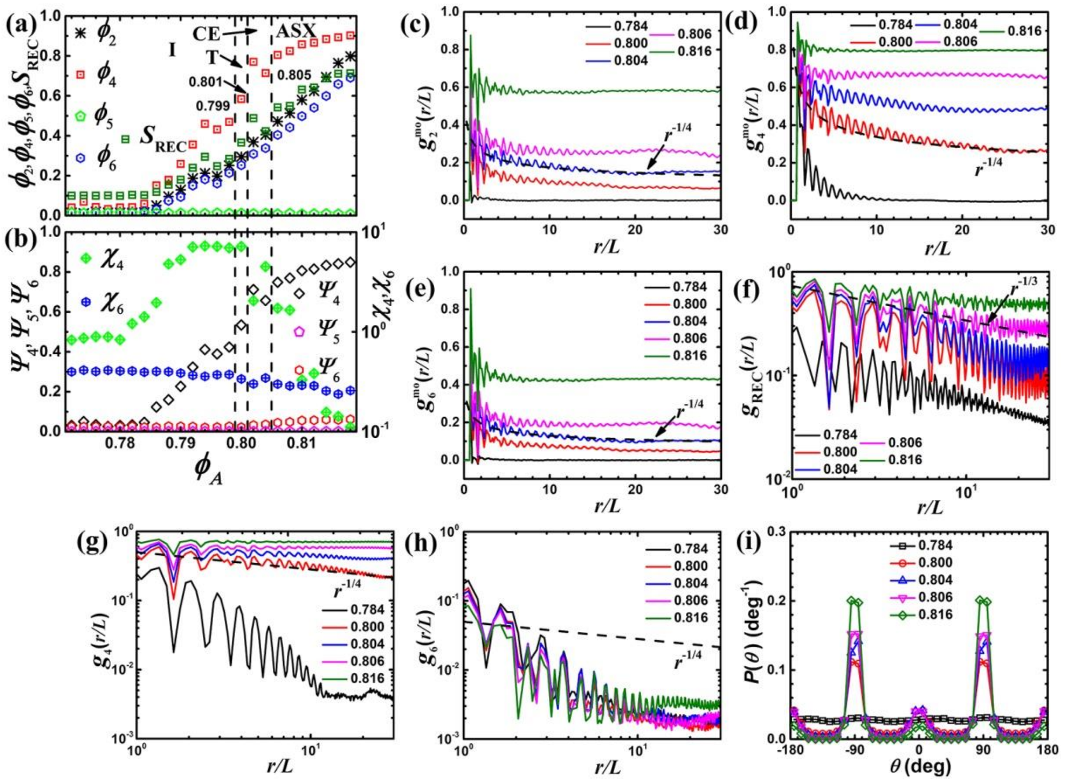

Supplementary Figure 25. Order parameters and correlation functions for different phases of kites of α = 112° obtained by *NVT* simulations. (a) Molecular-orientational order parameters, ϕ_2, ϕ_4, ϕ_5, and ϕ_6, positional order parameters of rectangular lattice (complex lattice) S_{REC}; (b) Bond-orientational order parameters, Ψ_4, Ψ_5 and Ψ_6, and susceptibilities of bond-orientational order parameters χ_4 and χ_6. Vertical dashed-lines are used to delimit different phases; (c-e) Molecular-orientational correlation functions, $g_2^{mo}(r)$, $g_4^{mo}(r)$ and $g_6^{mo}(r)$; (f) Spatial correlation functions of rectangular lattice, $g_{REC}(r)$; (g-h) Bond-orientational correlation functions, $g_4(r)$ and $g_6(r)$; (i) Distribution $P(\theta)$ of single-particle orientation. Dashed lines in (c-h) are curves $\propto r^{-1/4}$ or $r^{-1/3}$.

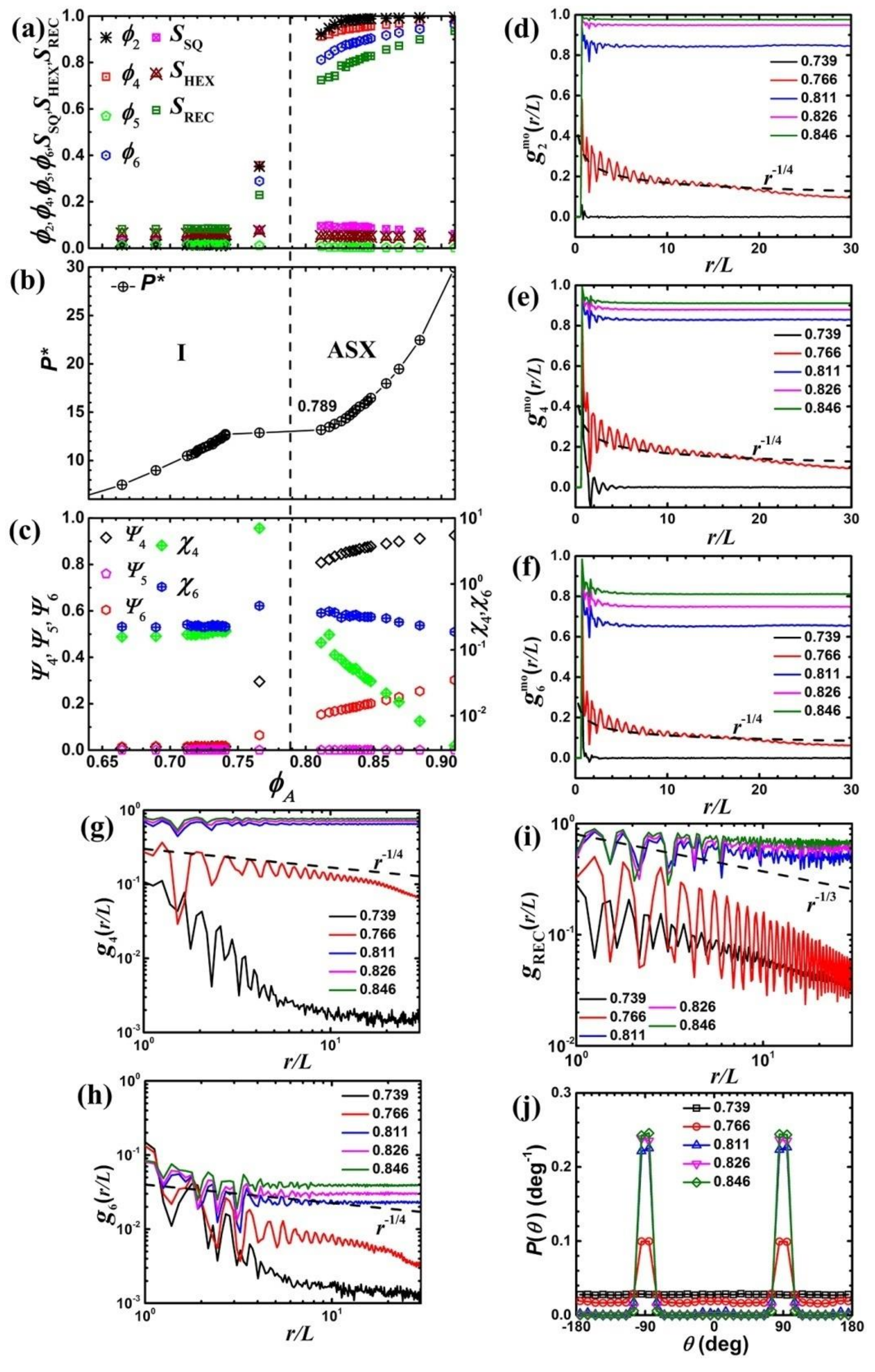

Supplementary Figure 26. Order parameters and correlation functions for different phases of kites of α = 126° obtained by *NPT* simulations. (a) Molecular-orientational order parameters, ϕ_2, ϕ_4, ϕ_5, and ϕ_6, positional order parameters of square lattice S_{SQ}, hexagonal lattice S_{HEX}, and rectangular lattice (complex lattice) S_{REC}; (b) Equation of state (EOS) with reduced pressure $P^* = PL^2/k_BT$; (c) Bond-orientational order parameters, Ψ_4, Ψ_5 and Ψ_6, and

susceptibilities of bond-orientational order parameters χ_4 and χ_6. Vertical dashed-lines are used to delimit different phases; (d-f) Molecular-orientational correlation functions, $g_2^{mo}(r)$, $g_4^{mo}(r)$ and $g_6^{mo}(r)$; (g-h) Bond-orientational correlation functions, $g_4(r)$ and $g_6(r)$; (i) Spatial correlation function of rectangular lattice, $g_{REC}(r)$; and (j) Distribution $P(\theta)$ of single-particle orientation. Dashed lines in (d-i) are curves $\propto r^{-1/4}$ or $r^{-1/3}$.

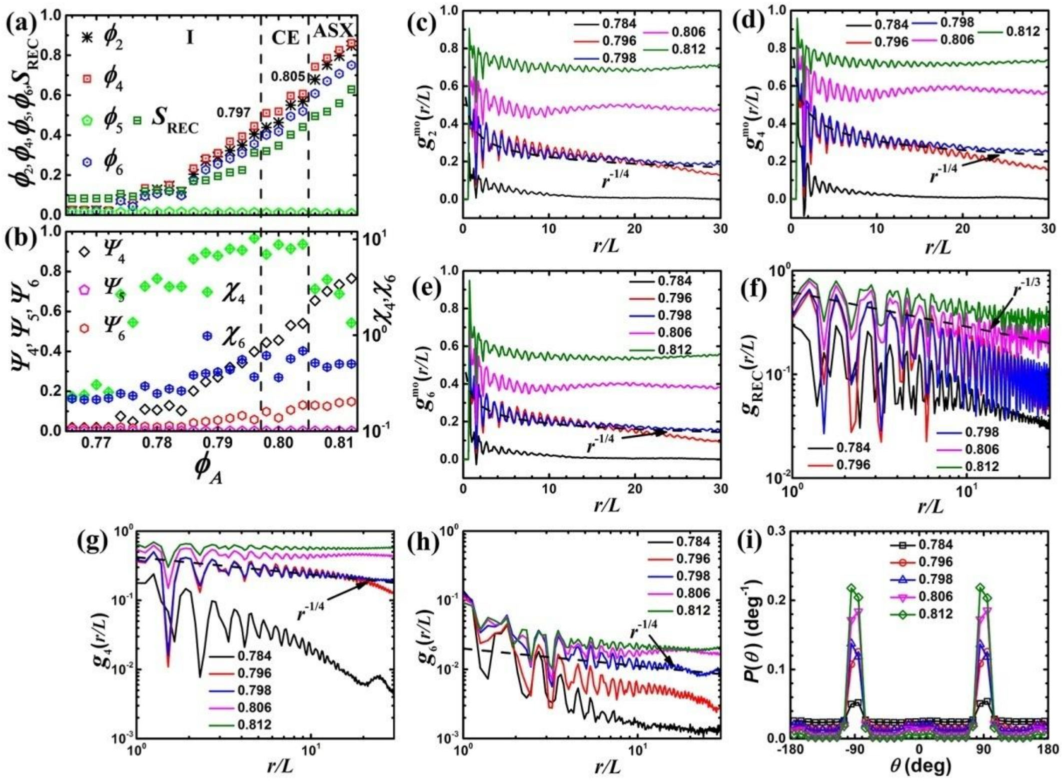

Supplementary Figure 27. Order parameters and correlation functions for different phases of kites of α = 126° obtained by *NVT* simulations. (a) Molecular-orientational order parameters, ϕ_2, ϕ_4, ϕ_5, and ϕ_6, positional order parameters of rectangular lattice (complex lattice) S_{REC}; (b) Bond-orientational order parameters, Ψ_4, Ψ_5 and Ψ_6, and susceptibilities of bond-orientational order parameters χ_4 and χ_6. Vertical dashed-lines are used to delimit different phases; (c-e) Molecular-orientational correlation functions, $g_2^{mo}(r)$, $g_4^{mo}(r)$ and $g_6^{mo}(r)$; (f) Spatial correlation functions of rectangular lattice, $g_{REC}(r)$; (g-h) Bond-orientational correlation functions, $g_4(r)$ and $g_6(r)$; (i) Distribution $P(\theta)$ of single-particle orientation. Dashed lines in (c-h) are curves $\propto r^{-1/4}$ or $r^{-1/3}$.

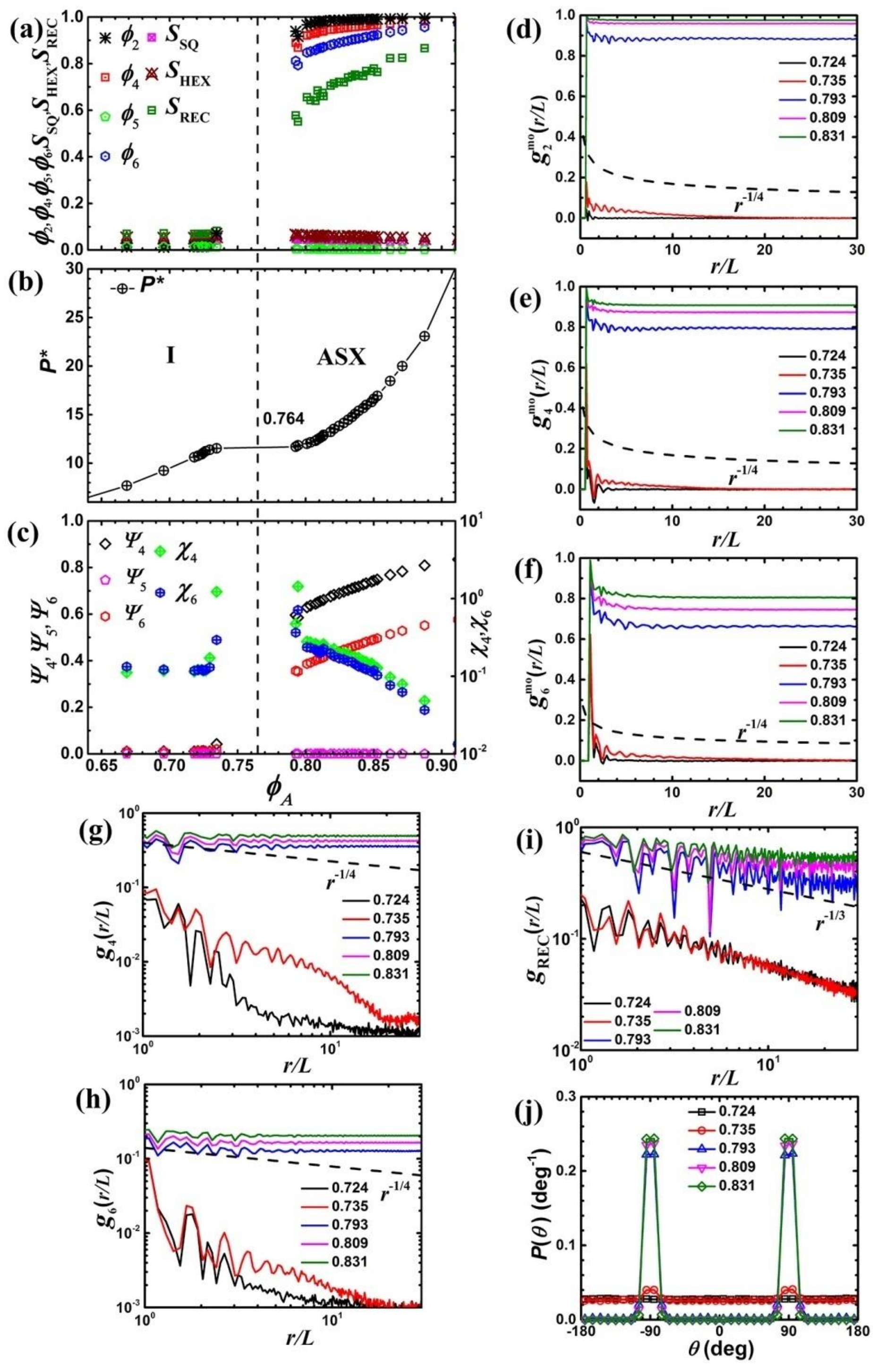

Supplementary Figure 28. Order parameters and correlation functions for different phases of kites of α = 144° obtained by *NPT* simulations. (a) Molecular-orientational order parameters, ϕ_2, ϕ_4, ϕ_5, and ϕ_6, positional order parameters of square lattice S_{SQ}, hexagonal lattice S_{HEX}, and rectangular lattice (complex lattice) S_{REC}; (b) Equation of state (EOS) with reduced pressure $P^* = PL^2/k_BT$; (c) Bond-orientational order parameters, Ψ_4, Ψ_5 and Ψ_6, and

susceptibilities of bond-orientational order parameters χ_4 and χ_6. Vertical dashed-lines are used to delimit different phases; (d-f) Molecular-orientational correlation functions, $g_2^{mo}(r)$, $g_4^{mo}(r)$ and $g_6^{mo}(r)$; (g-h) Bond-orientational correlation functions, $g_4(r)$ and $g_6(r)$; (i) Spatial correlation function of rectangular lattice, $g_{REC}(r)$; (j) Distribution $P(\theta)$ of single-particle orientation. Dashed lines in (d-i) are curves $\propto r^{-1/4}$ or $r^{-1/3}$.

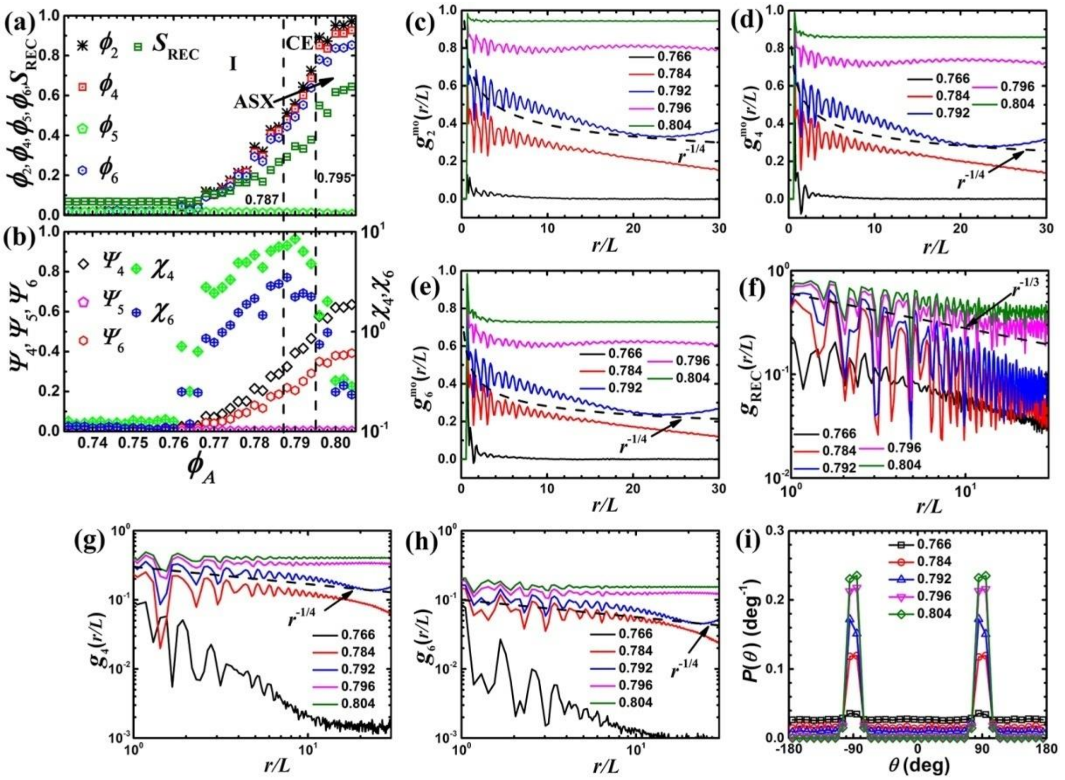

Supplementary Figure 29. Order parameters and correlation functions for different phases of kites of α = 144° obtained by *NVT* simulations. (a) Molecular-orientational order parameters, ϕ_2, ϕ_4, ϕ_5, and ϕ_6, positional order parameters of rectangular lattice (complex lattice) S_{REC}; (b) Bond-orientational order parameters, Ψ_4, Ψ_5 and Ψ_6, and susceptibilities of bond-orientational order parameters χ_4 and χ_6. Vertical dashed-lines are used to delimit different phases; (c-e) Molecular-orientational correlation functions, $g_2^{mo}(r)$, $g_4^{mo}(r)$ and $g_6^{mo}(r)$; (f) Spatial correlation functions of rectangular lattice, $g_{REC}(r)$; (g-h) Bond-orientational correlation functions, $g_4(r)$ and $g_6(r)$; (i) Distribution $P(\theta)$ of single-particle orientation. Dashed lines in (c-h) are curves $\propto r^{-1/4}$ or $r^{-1/3}$.

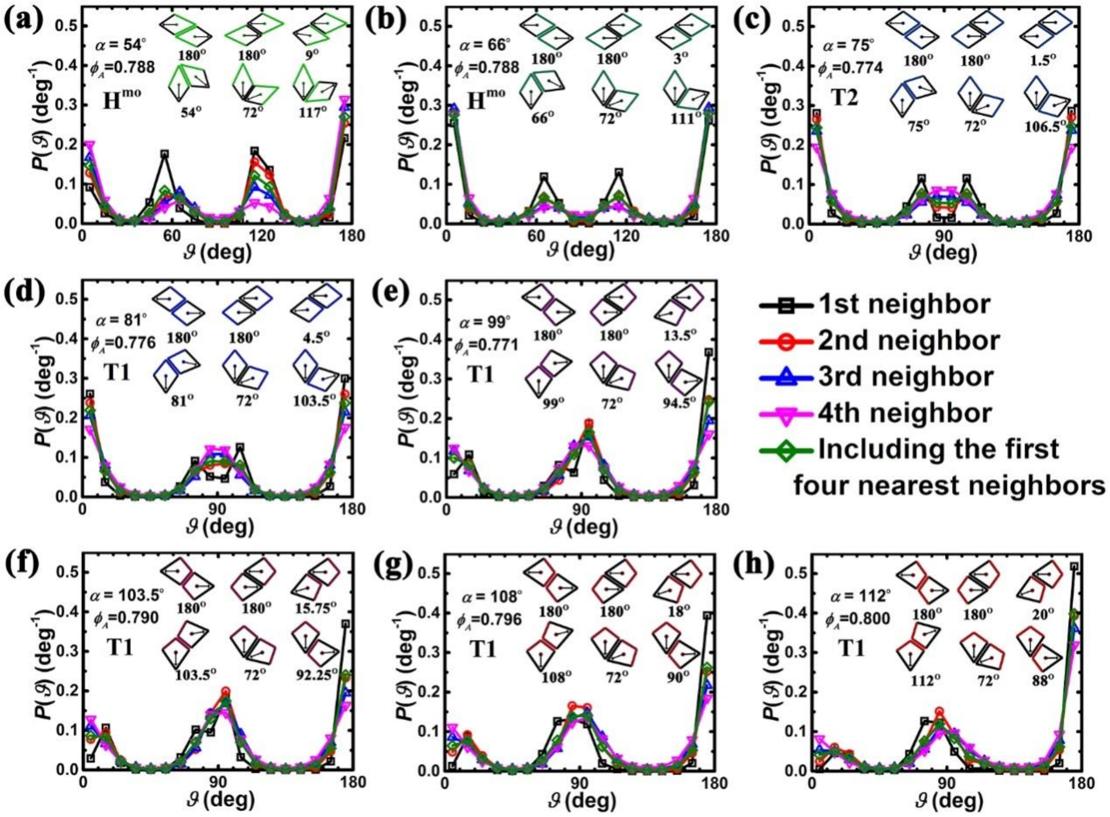

Supplementary Figure 30. Relative pointing angle distribution $P(\vartheta)$. (a-b) $P(\vartheta)$ in a hexatic phase formed by kites of α = 54° and 66°, respectively; (c) $P(\vartheta)$ in a tetratic phase T2 formed by kites of α = 75°; (d-h) $P(\vartheta)$ in a tetratic phase T1 formed by kites of α = 81°, 99°, 103.5° , 108° and 112°, respectively. The distributions of the first, second, third, fourth and the total four nearest neighbors are shown. Insets illustrate LPC-NPPs with associated relative pointing angles for each type of kite.

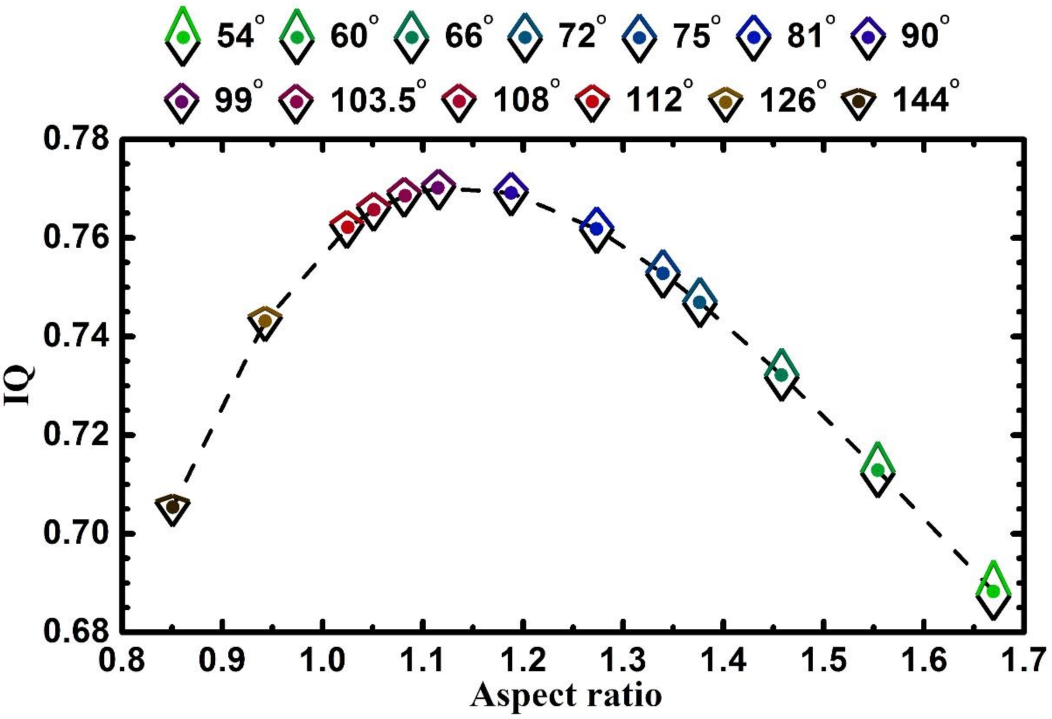

Supplementary Figure 31. Aspect ratio (L_l / L_t) and isoperimetric quotient (IQ) of kites.

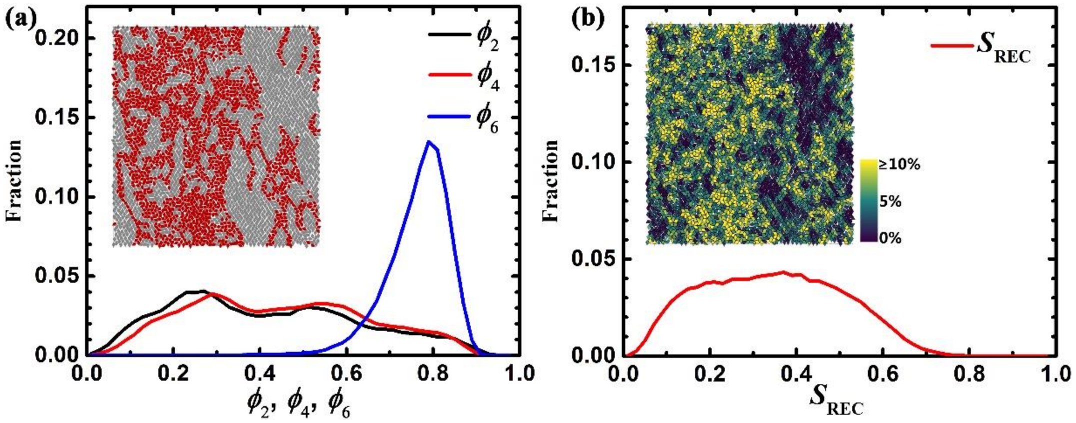

Supplementary Figure 32. Histograms of order parameters. (a) Histograms of local *n*-fold molecular-orientational order parameters. (b) A histogram of local positional order parameter (rectangular lattice) of a representative CE region of H^{mo} and ASX structures observed in kites of $\alpha = 60°$ at $\phi_A = 0.798$. Local order parameters of a particle are calculated over a circular region with a radius of 5L centered at the interested particle. The histogram of ϕ_6 shows one peak as both H^{mo} and ASX structures have high ϕ_6 order parameter. The histograms of ϕ_2 and ϕ_4 show two weak broad peaks with one peak at moderate order parameters and the other at low order parameters. By contrast, the histogram of S_{REC} shows a broad plateau-like curve. Rotationally, for each particle, we can find its neighbors using Voronoi construction, and then calculate the relative pointing angles between this particle and its neighbors. If there are more than half of its neighbors whose relative pointing angles are not in the vicinity of 180° and 0°, this particle is called to be rotated particles and likely not belong to ASX structures. Inset in (a) shows a colored configuration in the representative CE region, in which red particles are rotated particles. Positionally, we can first calculate the IQ of Voronoi tessellation of each particle, and then get the deviation of IQ relative to the corresponding value of a perfect ASX crystal of the same surface fraction. Inset in (b) shows the same configuration as in the inset in (a) but is colored by such IQ deviations. The patch-like patterns shown in the two insets are consistent with each other, and both support the coexistence of two different structures. It is also noticed that there are many small patches in the configuration whose S_{REC} can have large fluctuations due to relatively small numbers of particles in

each patch, which then can contribute to the resulted broad plateau-like histogram of S_{REC}.

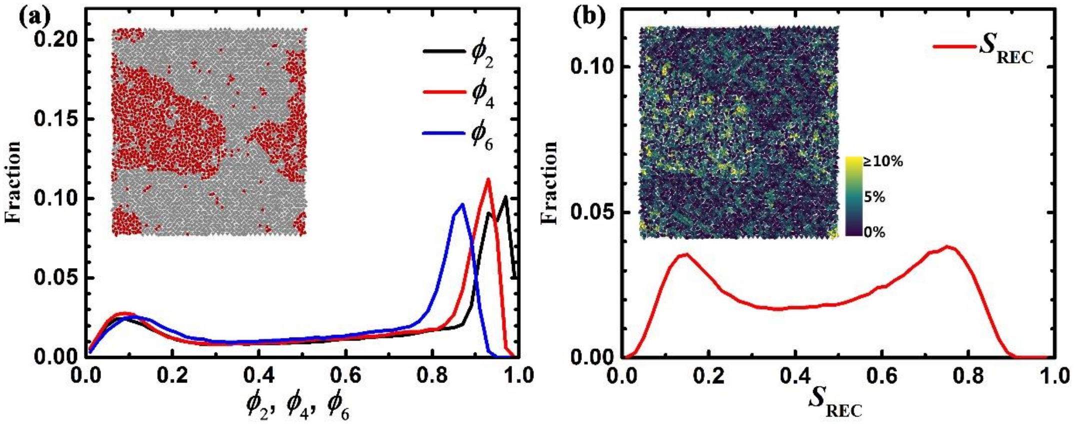

Supplementary Figure 33. Histograms of order parameters. (a) Histograms of local *n*-fold molecular-orientational order parameters. (b) A histogram of local positional order parameter (rectangular lattice) of a representative CE region of isotropic and ASX structures observed in kites of α =144° at ϕ_A = 0.792. Local order parameters of a particle are calculated over a circular region with a radius of 5*L* centered at the interested particle. Two peaks with one at high order parameters and the other at low order parameters are observed, indicating the coexistence of disordered isotropic structures and ordered ASX structures. By performing similar color-coding as in Supplementary Figure 32, inset in (a) shows a colored configuration in the representative CE region, in which red particles are rotated particles. And inset in (b) shows the same configuration but colored by IQ deviations. The patch-like patterns shown in the two insets are consistent with each other, and both support the coexistence regions of isotropic and ASX structures as evidenced by the histograms of order parameters.

Supplementary References

1. Frenkel, D. & Smit, B. Understanding molecular simulation: From algorithms to applications (Academic Press, 1996).
